# Integrated genomic analysis and CRISPRi implicates *EGFR* in Alzheimer’s disease risk

**DOI:** 10.1038/s44400-025-00049-5

**Published:** 2025-12-16

**Authors:** Yuk Yee Leung, Pavel P. Kuksa, Luke Carter, Jeffrey Cifello, Emily Greenfest-Allen, Otto Valladares, Louisa Boateng, Shannon Laub, Natalia Tulina, Sofia Moura, Aura Ramirez, Katrina Celis, Fulai Jin, Ru Feng, Gao Wang, Phil De Jager, Jeffery M. Vance, Liyong Wang, Struan F. A. Grant, Gerard D. Schellenberg, Alessandra Chesi, Li-San Wang

**Affiliations:** 1 Penn Neurodegeneration Genomics Center, Department of Pathology and Laboratory Medicine, Perelman School of Medicine, University of Pennsylvania, Philadelphia, PA USA; 2 John P. Hussman Institute for Human Genomics, University of Miami Miller School of Medicine, Miami, FL USA; 3 Department of Genetics and Genome Sciences, School of Medicine, Case Western Reserve University, Cleveland, OH USA; 4 Center for Statistical Genetics, The Gertrude H. Sergievsky Center, Columbia University, New York, NY USA; 5 Department of Neurology, Columbia University, New York, NY USA; 6 Center for Spatial and Functional Genomics, The Children’s Hospital of Philadelphia, Philadelphia, PA USA; 7 Divisions of Human Genetics and Endocrinology & Diabetes, The Children’s Hospital of Philadelphia, Philadelphia, PA USA; 8 Department of Genetics, Perelman School of Medicine, University of Pennsylvania, Philadelphia, PA USA; 9 Department of Pediatrics, Perelman School of Medicine, University of Pennsylvania, Philadelphia, PA USA

**Keywords:** Computational biology and bioinformatics, Genetics, Neurology, Neuroscience

## Abstract

Genome-wide association studies (GWAS) have identified numerous loci linked to late-onset Alzheimer’s disease (LOAD), but the pan-brain regional effects of these loci remain largely uncharacterized. To address this, we systematically analyzed all LOAD-associated regions reported by Bellenguez et al. using the FILER functional genomics catalog across 174 datasets, including enhancers, transcription factors, and quantitative trait loci. We identified 41 candidate causal variant-effector gene pairs and assessed their impact using enhancer–promoter interaction data, variant annotations, and brain cell-type-specific gene expression. Notably, the LOAD risk allele of rs74504435 at the *SEC61G* locus was computationally predicted to increase *EGFR* expression in LOAD-related cell types: microglia, astrocytes, and neurons. Functional validation using promoter-focused Capture C, ATAC-seq, and CRISPR interference in the HMC3 human microglia cell line confirmed this regulatory relationship. Our findings reveal a microglial enhancer regulating *EGFR* in LOAD, suggesting *EGFR* inhibitors as a potential therapeutic avenue for the disease.

## Introduction

Alzheimer’s disease (AD) is the leading cause of dementia in the United States and currently lacks effective treatments or prevention strategies. The most common form, late-onset Alzheimer’s disease (LOAD), typically begins after age 60 and is highly heritable (60-80%), indicating a significant genetic component in its development^1^. While the *APOE* locus remains the strongest genetic risk factor^2^, LOAD is complex and highly polygenic^3^. Previous genome-wide association studies (GWAS) identified over 20 LOAD-associated loci^2,4^; recent studies using UK Biobank proxy-AD or proxy-control samples have expanded this list to 75 loci^5,6^. Although progress has been made in linking LOAD genetic risk to microglial-mediated innate immune processes^7–9^, the broader cellular contexts of these variants remain incompletely understood. Emerging evidence suggests that LOAD-associated variants also affect other brain cell types, including myeloid cells^10^, astrocytes, and neurons, but the mechanisms across these diverse cellular environments remain largely uncharacterized.

Over 90% of GWAS variants are located in non-coding regions of the genome, outside of protein-coding sequences^10,11^. These non-coding variants are widely hypothesized to affect gene regulatory elements, such as enhancers^12–14^, which can influence the expression of distant target genes^15^. The difficulty in identifying such distal genes arises from the challenges related to linkage disequilibrium (LD) with nearby non-causal variants and the variability in the biological contexts of the corresponding target ‘effector’ genes. Despite these, some studies have applied various statistical and computational methods, along with new data types, to analyze non-coding GWAS signals for AD^10,16–18^. This effort is important because drugs with genetic support are twice as likely to gain approval^19–21^.

However, these studies face limitations in their analytical strategies. First, they traditionally focus only on top GWAS signals (sentinel variants), restricting the understanding of AD’s full genetic landscape^5,6,16^. Since any variant in LD with a sentinel could be causal, comprehensive analyses should include both sentinel and nearby LD variants. Second, genome-wide functional genomics data for brain tissues or specific cell types remain limited and relatively small compared to data from other cell types or cell lines^22–24^. Third, prior computational analyses of non-coding variants often failed to integrate eQTLs from independent sources to confirm consistency and replication of effect alleles and signal direction^5,6,10,11^. Finally, no previous analyses have combined eQTLs and enhancer–promoter interactions (EPI) to prioritize variant-to-gene (V2G) pairs in LOAD.

To address these limitations, we developed an enhanced post-GWAS non-coding variant analysis framework^25^. This approach systematically identifies candidate causal variants and relevant regulatory genomic features to improve our understanding of genetic loci associated with LOAD.

## Results

**Fig. 1:**
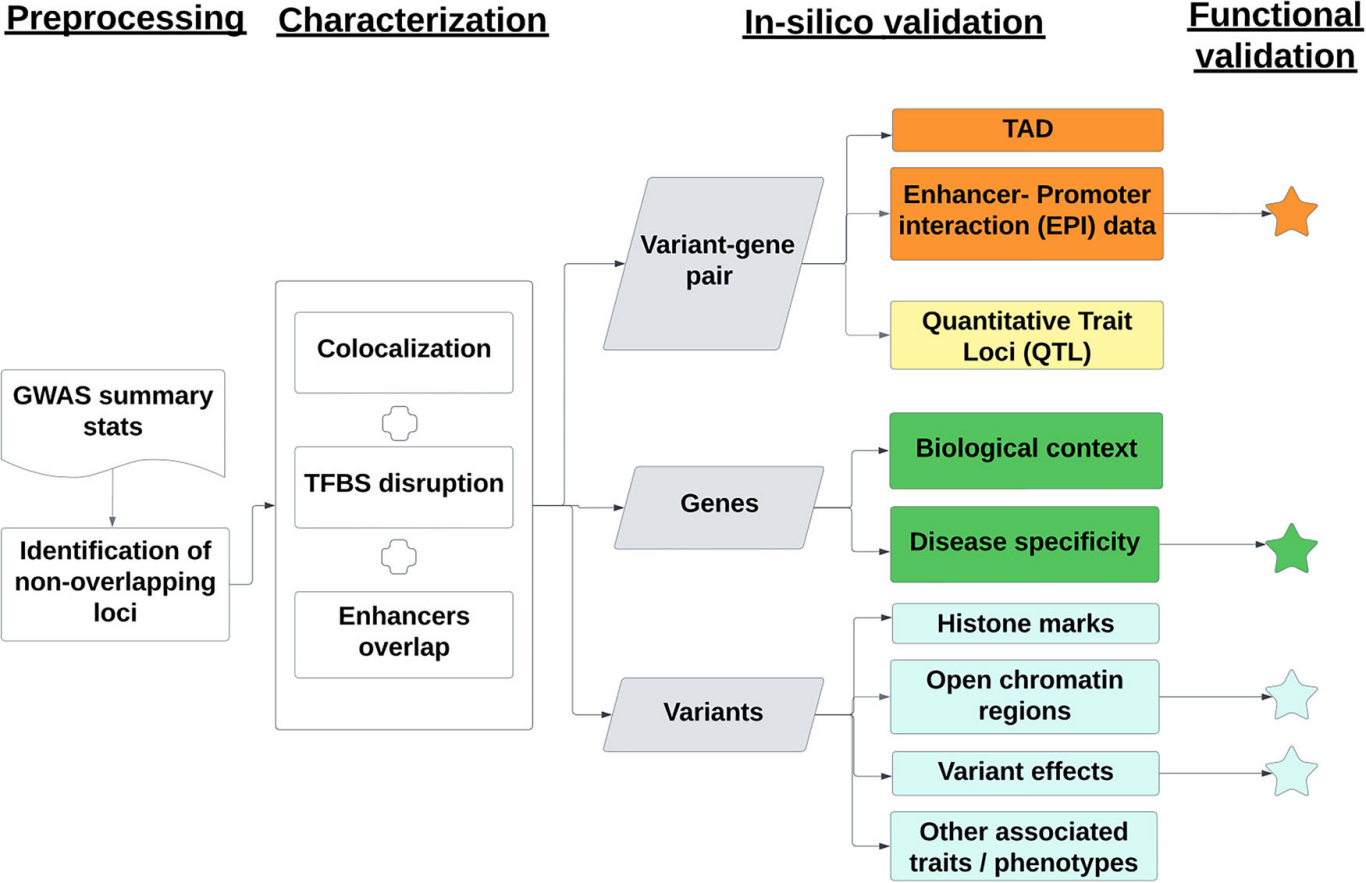
Analysis strategy. Our post-GWAS framework consists of the following steps: “Preprocessing,” “Characterization,” “In silico validation,” and “Functional validation.” Genome-wide significant variants from Bellenguez et al.^5^ were leveraged to identify regions of interest. Putative causal variants were analyzed for functional contexts and linked to potential causal genes using enhancers, eQTL data, and TFBS predictions. These variant-to-gene (V2G) pairs were characterized in brain tissues and cell types. Independent assays, including enhancer–promoter interactions (EPI), QTLs, and open chromatin regions from new data sources, were used for in silico validation. Functional validation included promoter-focused Capture C, ATAC-seq, RNA-seq, and CRISPRi in microglial cells.

We summarize our strategy in Fig. 1, which comprises four key steps: “Preprocessing,” “Characterization,” “In silico validation,” and “Functional validation”.

1. In the “Preprocessing**”** stage, we obtained pairwise-independent tag variants through LD-based pruning (1000 Genome panel) on all genome-wide significant LOAD GWAS variants (*p* < 5 × 10^−^^8^)^5^. To produce a larger pool of candidate causal variants, we included all proxy variants through LD-based expansion of the tag variant set (*r*^2^ > =0.7).
2. In the “Characterization” step, these variants were annotated using FILER^26^ (a large-scale genomic data queryr tool), and their potential functional context(s) were predicted using SparkINFERNO^25^ (a scalable pipeline for inferring non-coding variants' molecular mechanisms). We identified candidate causal genes per variant using eQTL data. Additionally, we used HOMER^27^ to predict transcription factor binding site (TFBS) disruptions caused by the variants. Only variants within brain enhancers were retained in subsequent analysis steps. Together with their corresponding genes, this set formed the enhancer-based causal V2G pairs with TFBS.
3. For the “In silico validation” step, we annotated and ranked the V2G pairs, putative causal variants, and effector genes using independent assays from new data sources. These include regulatory features (EPIs, QTLs, and open chromatin regions) and expression datasets (bulk RNA-seq, proteomics). We also performed consistency checks on QTLs across data sources.
4. Finally, in the “Functional validation” step, we specifically contextualized one V2G pair using our existing datasets from multiple cell types, and in vitro validation using CRISPR interference (CRISPRi).

Overall, we defined context-specific regulatory elements (variants and genes) across different cellular and tissue contexts, leveraging 174 datasets from 10 data sources. The number of regions, variants, and genes identified in each of the 4 steps in Fig. 1 is summarized in Supplementary Data 1. This enabled independent in silico validation of 41 variant-effector-gene (V2G) pairs. The rs74504435-*EGFR* V2G pair underwent further functional characterization, confirming it as a therapeutically tractable target.

### Preprocessing: identification of genomic regions and candidate variants of interest

To define regions of interest and establish an initial discovery set of plausible candidate variants for further analyses, we leveraged the set of 5586 genome-wide significant variants (*p* < 5 × 10^−^^8^) from the full GWAS summary statistics^5^. We performed LD pruning using the 1000 genomes EUR panel^28^, obtaining 580 pairwise-independent (tag) variants (*r*² < 0.7). For each tag variant, we expanded its region of interest to include all proxy variants (*r*² ≥ 0.7) within 1 Mbps, with boundaries set by the most distant proxies. This LD-based expansion yielded a total of 9144 plausible candidate variants across all identified regions of interest (*Preprocessing in* Fig. 1*,* *Methods: Preprocessing*), increasing the candidate pool by 64% (from 5586 to 9144). Most candidate variants were non-coding, predominantly intergenic (38%) or intronic (24%), with 35% located in 5′ and 3′ UTR introns (Supplementary Fig. 1).

### Characterization: identification of putative causal variants, genes, and variant-gene pairs

To explore the regulatory roles of candidate variants, we adapted the SparkINFERNO framework^25^ and overlapped variants with 174 brain-related functional genomics (FG) tracks in FILER^26^. These tracks cover 35 brain regions and 7 brain cell types across 10 data resources, representing five regulatory types: enhancers, histone modifications, QTLs, EPIs, and topologically associating domains (TADs) (Supplementary Fig. 2).

**Fig. 2:**
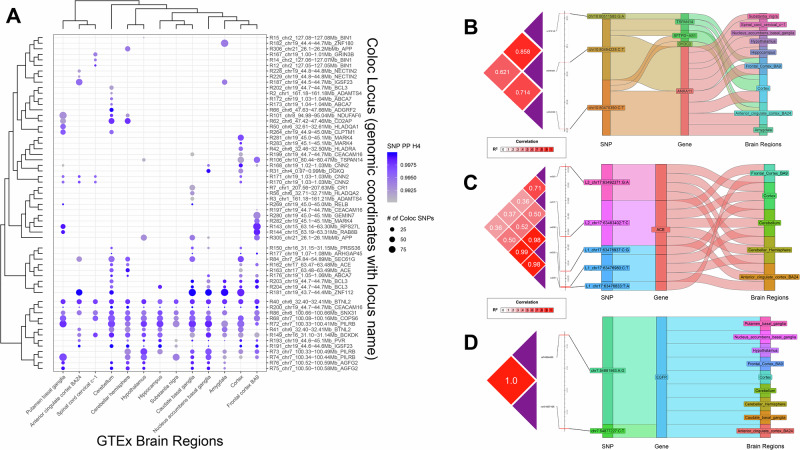
Colocalization results. **A** A subset of significant colocalization results across 59 loci and 13 GTEx brain regions. **B** All significant brain colocalization results of *TSPAN14*. **C** Significant brain colocalization results from multiple independent loci inside the *ACE* region. **D** All significant brain colocalization results of *EGFR*.

We quantified the probability of a candidate regulatory variant co-localizing with an eQTL signal. Among 9144 variants (including 1355 on chr19 in the APOE region), 229 had at least one colocalized signal (locus-level posterior colocalization probability (PP.H4.abf) >0.7), individual SNP-level posterior colocalization probability (SNP.PP.H4 > 0.5; see Supplementary Data 2 for the summary of colocalization results when using more stringent PP.H4.abf thresholds) in any of 13 GTEx^29^ brain tissue-specific eQTL tracks (see Supplementary Data 3 for the colocalized signals with FDR < 0.05, i.e., PP.H4.abf >0.95). These 229 variants defined 1601 tissue-V2G pairs, putatively regulating 232 genes. Notably, 14 variants were identified in ≥10 brain regions, while 103 variants (44.9%) were brain-region specific. Figure 2A highlights a subset of the top colocalization results (defined by locus-level posterior colocalization probability (PP.H4.abf) >0.99 and fewer than 100 SNPs per locus) across GTEx brain tissues.

A single line of functional evidence is often considered insufficient to robustly implicate regulatory activity at a given locus. To address this limitation, we developed an unbiased confluent context identification strategy (*Methods: “Steps for unbiased confluent context identification”*) to integrate multiple lines of evidence to prioritize putative causal variants, genes, and V2G pairs for subsequent experimental validation. Of the 229 colocalized candidate variants, 68 (29.69%) overlap predicted HOMER^27^ TFBS (*Method: Transcription factor binding site (TFBS) disruption*). Among these, 15 (22%) and 23 (34%) fall within brain enhancers defined by ROADMAP^30^ and EpiMAP^31^, respectively (*Method: Enhancers overlap*). Overall, we identified 24 putatively causal variants (14 beyond the *APOE* locus) overlapping a brain eQTL, a brain enhancer (ROADMAP or EpiMAP), and a TFBS, forming 279 candidate tissue-V2G pairs involving 32 potential effector genes. Among non-*APOE* variants, 36% colocalized with >50% of GTEx brain tissues, with each variant interacting with an average of three (maximum 11) candidate effector genes. Notably, seven non-*APOE* variants did not affect the annotated (typically closest) genes in the original GWAS (Table 1)^5^. All selected colocalized candidate variants were common (non-reference allele frequency >0.05 in GWAS and 1000G). Table 1 summarizes the 14 candidate regulatory variants (beyond *APOE*) identified by this unbiased confluent context identification approach (see Supplementary Data 4 for robustness/sensitivity analysis of our V2G set under different/more stringent LD pruning thresholds). All eQTL effector genes and coloc results are detailed in Supplementary Data 5.

**Table 1:** Genome-wide analysis of AD GWAS data implicates 14 candidate regulatory variants (outside chr19/*APOE*) with potential roles in brain tissue using the confluent context identification approach

From GWAS				Colocalization		Enhancers		TFBS	
Variant	rsID	Loci	Closest gene	Causal genes	Brain regions	ROADMAP	EpiMAP	HOMER	Motif logo
chr1:161186243:C:A	rs11585858	Not reported	ADAMTS4	2	3	6	32	GLIS3	
chr1:161189357:C:T	rs4233366	Not reported	ADAMTS4	1	1	0	1	PAX6	
chr7:100242838:A:G	rs866500	Not reported	PVRIG	11	12	6	13	GLIS3	
chr7:100373690:T:C	rs2405442	ZCWPW1/NYAP1	PILRA	7	10	2	8	ERRA	
chr7:100374211:A:G	rs1859788	ZCWPW1/NYAP1	PILRA	5	4	2	6	ERRA	
chr7:100561944:A:G	rs2734897	Not reported	AGFG2	12	12	0	1	PPARE	
chr7:143410495:G:T	rs12703526	EPHA1	EPHA1	1	1	3	10	SCL	
chr7:54881563:A:G	rs74504435	SEC61G	SEC61G	1	2	3	4	OCT6	
chr8:27354759:A:C	rs10109834	PTK2B	PTK2B	1	2	0	1	NANOG	
chr16:31122128:C:T	rs1060506	KAT8	KAT8	1	1	0	1	SMAD2	
chr16:31143037:G:A	rs78924645	Not reported	PRSS36	3	9	0	1	C-MYC	
chr16:81739604:A:G	rs12444183	PLCG2	PLCG2	1	1	0	1	TATA-BOX	
chr17:63492371:G:A	rs4351	ACE	ACE	3	5	0	1	PRDM10	
chr21:26161943:T:C	rs4817090	APP	APP	3	12	6	6	CUX2	

To further explore the genetic architecture of gene regulation in the brain, we examined loci with multiple candidate causal variants and their effects on gene expression across brain regions. Figure 2B highlights the significant colocalization results for the *TSPAN14* locus, where three candidate causal variants were associated with the expression of four genes across nine GTEx brain regions. Figure 2C shows three independent signals, L1, L2, and L3 (pairwise *r*^2^ = 0.501, 0.364, and 0.709, respectively) at the *ACE* locus, each correlating with *ACE* gene expression across five GTEx brain regions. Figure 2D shows the two variants (*r*^2^ = 1.00) inside the *EGFR* locus. Together, these figures illustrate the complexity and diversity of genetic regulation, demonstrating how multiple independent variants can influence gene expression across diverse brain regions.

### Potential biological roles of the identified transcription factor binding motifs

We found that the candidate regulatory variants are predicted to disrupt binding sites for 12 transcription factors (TFs) based on PWM analyses (Table 1). Several of these TFs are involved in mechanisms related to AD. Notably, GLIS3 (GLIS family zinc finger 3) is the only motif strongly linked to tau and amyloid pathology through transcriptional regulation^32–34^. SMAD2 (Mothers against decapentaplegic homolog 2), a key intracellular protein in the SMAD family, transduces signals from TGF-β ligands and mediates cellular responses. The TGF-β/SMAD2 pathway plays a complex role in AD, potentially affecting cell growth, differentiation, and immune responses^35^. Dysfunction in TGF-β signaling may lead to blood-brain barrier breakdown, and blocking TGF-β-SMAD2/3 signaling in peripheral macrophages has been proposed as a therapeutic strategy for AD^36^.

### In silico validation: identification of putative causal signals with further support

Outside the APOE region, we identified 41 candidate V2G pairs comprising 14 variants and 26 protein-coding genes that are likely functional in brain tissues or cell types. For additional in silico support, we leveraged independent FG datasets. Recent FG data from large consortia^37^, harmonized studies^38–41^, and individual publications^42,43^ provided complementary or orthogonal evidence for in silico validation. We processed these datasets using hipFG^44^ and integrated them into FILER^26^, including two QTL (MetaBrain^39^, eQTL Catalogue^45^) and three EPI datasets (3DGenome^41^, 4DGenome^40^, Nott et al.^43^), standardizing all with metadata for efficient querying.

We first assessed the consistency of V2G pairs identified in brain regions across different data sources. Consistency in directionality means that a genetic variant’s effect on an eQTL (i.e., increasing or decreasing target gene expression) is the same across brain regions and data sources. Such consistency suggests shared genetic regulatory mechanisms across brain regions. However, eQTL directionality, represented by Z-scores, can vary between datasets due to (a) sampling differences, (b) QTL generation methods, or (c) statistical approaches. Since QTL datasets lack standardized presentation, harmonizing them with tools like hipFG^44^ can reduce these biases and improve interpretation.

**Fig. 3:**
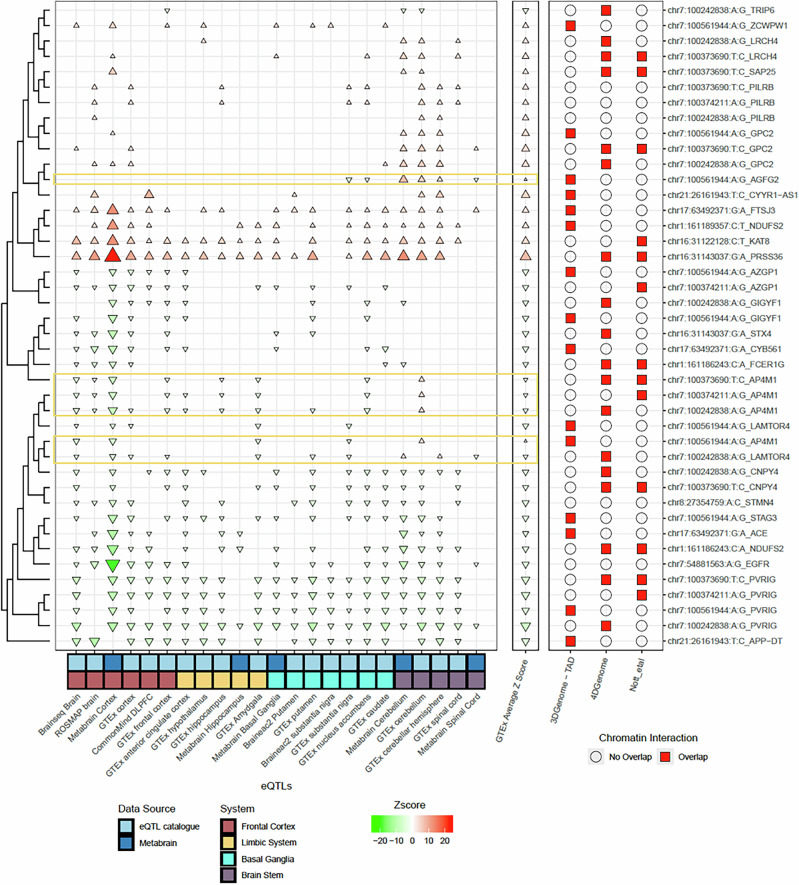
Comparison of the directionality of selected putative V2G pairs across QTL and EPI datasets used for in silico validation. All functional genomics data were processed using hipFG^44^, with effect directionality normalized. For both the left and middle panels, triangles pointing upwards (and in red) mean a positive Z-score, indicating that the alternative allele of a variant increases the gene expression, while those pointing downwards (and in green) mean the opposite effect. The size of the triangles represents the absolute value of the Z-score. The left panel shows the directionality (Z-scores) based on 23 QTL datasets from two data sources. The middle panel presents the average GTEx Z-scores, while the right panel displays the orthogonal support of EPI data for the V2G pairs (with a red square indicating the presence of EPI for that particular data source). The six loci with inconsistent directionalities were shown in yellow.

In Fig. 3, the left panel shows hipFG-normalized Z-scores for each brain region (“System” in legend: Frontal Cortex, Limbic System, Basal Ganglia, Brain Stem) from two data sources (MetaBrain or eQTL Catalogue), along with average Z-scores across 13 GTEx brain regions (middle panel). The original, non-normalized Z-scores (Supplementary Fig. 3**)** show that 35 out of 41 V2G pairs had inconsistent directionality over >50% of the grouped brain regions. Strikingly, after Z-score normalization, inconsistencies dropped to only 6, an 83% improvement (McNemar’s Test, *p* < 0.0001), as shown in Fig. 3 (left panel).

### A ranking system integrating in silico evidence on genes, variants, and V2G pairs for selected putative causal signals

When an eQTL and an EPI co-occur for a given variant-to-gene (V2G) pair, it provides stronger evidence that the variant causally regulates gene expression^46,47^. To prioritize V2G pairs, we developed a ranking system based on four components: eQTL evidence (V2G_eQTL tier, assesses consistency of V2G pairs across gene expression datasets), EPI evidence (V2G_EPI tier, checks for V2G pairs in brain EPIs), variant properties (V_tier, evaluates variant characteristics), and gene expression (G_tier, considers gene relevance and expression). Each tier was generated by integrating data from multiple sources (*Methods: Ranking of variant-gene pairs (Tier system)*. This structured approach helps prioritize V2G pairs based on robust, multi-source evidence.

**Fig. 4:**
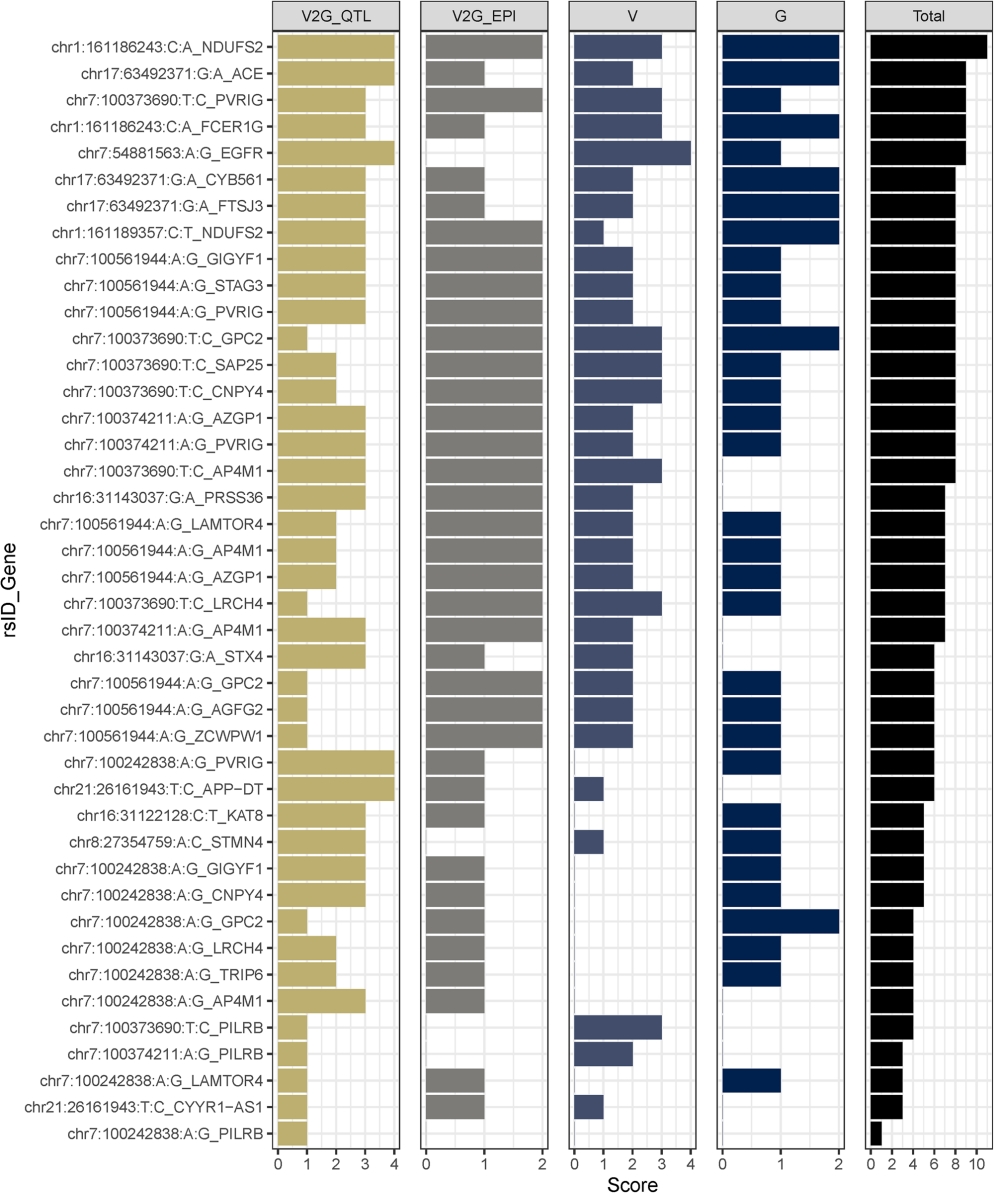
Rankings (V2G_eQTL tier, V2G_EPI tier, V_tier, G_tier, and overall tier) of the 42 V2G pairs identified in this study. To prioritize variant-to-gene (V2G) pairs for functional validation, we developed a ranking system based on four components: eQTL evidence (V2G_eQTL tier), EPI evidence (V2G_EPI tier), variant properties (V_tier), and gene expression (G_tier). Each tier was generated by integrating data from multiple sources.

The V2G_eQTL and V2G_EPI information are presented in Fig. 3, while overall tier rankings are displayed in Fig. 4. The top-ranked V2G pair was chr1:161186243:C:A_*NDUFS2* (tier 11), followed by four pairs at tier 9 (chr7:54881563:A:G_*EGFR*, chr1:161186243:C:A_*FCER1G*, chr7:100373690_*PVRIG*, and chr17:63492371:G:A_*ACE*). These five pairs are hypothesized to have the greatest likelihood of functional study success.

### Functional validation: an enhancer region harboring rs74504435 influences EGFR expression

**Fig. 5:**
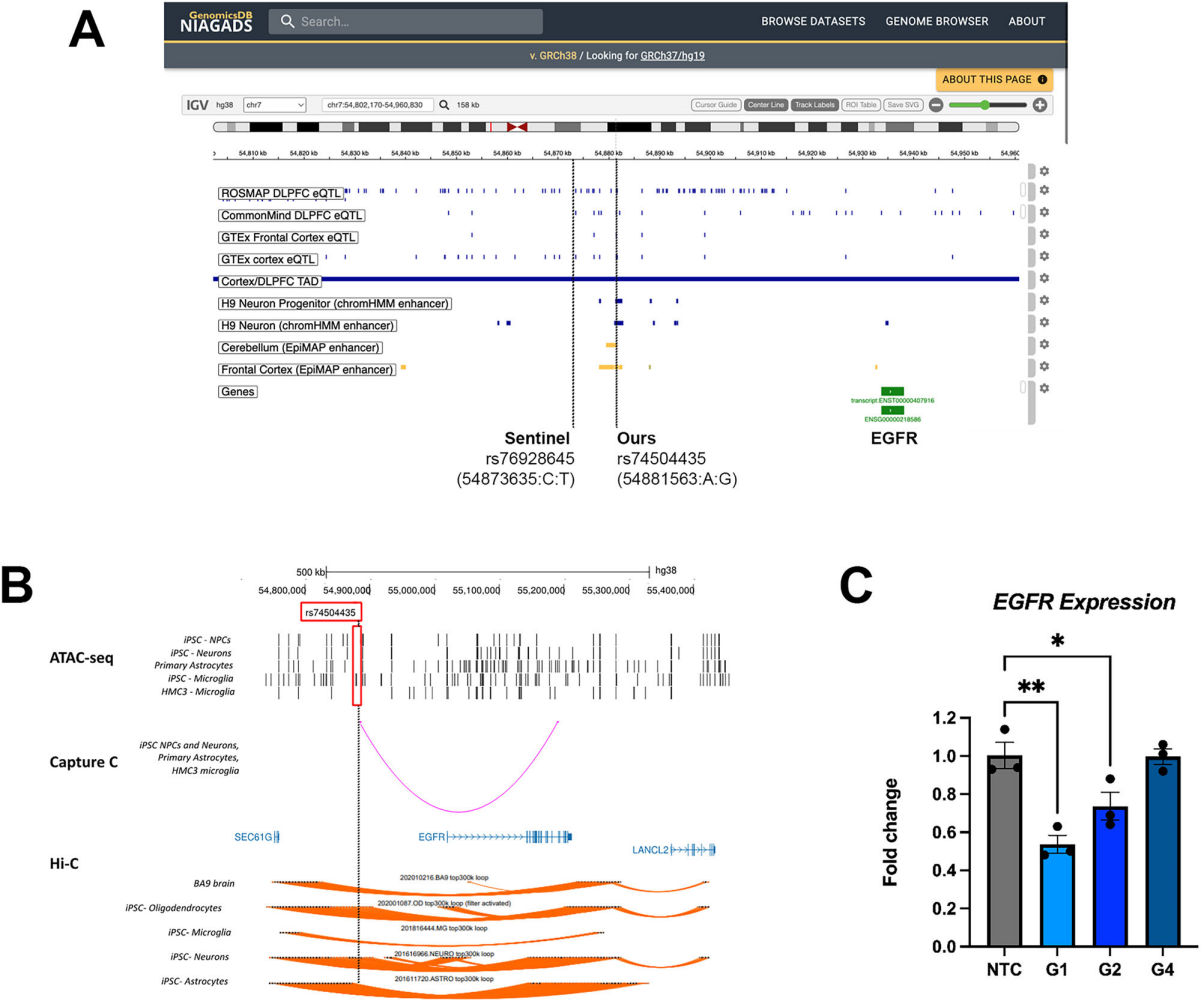
Functional annotation and follow-up for the selected V2G pair: chr7:54881563:A:G (rs74504435) and *EGFR.* **A** Genome browser plot showing the functional annotation of the selected V2G pair in GRCh38 using FILER tracks at the discovery phase, including enhancers from chromHMM and EpiMAP, eQTLs from GTEx and eQTL Catalogue. Bellenguez GWAS identified the sentinel SNP rs76928645 (chr7:54873635:C:T) (*p* = 1.6 × 10^−^^10^), in high LD (*r*^2^ = 0.94) with our variant of interest rs74504435, which is annotated to reside in a brain enhancer in four different data sources. Plot is generated using NIAGADS genomicsDB. **B** ATAC-seq, promoter-focused Capture C, and Hi-C data in brain-relevant cell types showing chromatin state and looping between chr7:54881563:A:G (rs74504435) and *EGFR*. rs74504435 (highlighted by a yellow line) resides in open chromatin in our own iPSC-derived neural progenitors, neurons, and microglia, and in primary astrocytes. It is involved in a chromatin loop with *EGFR* in our own iPSC-derived neurons, primary astrocytes, and the microglial cell line HMC3 (promoter-focused Capture C data); as well as in our own iPSC-derived neurons and oligodendrocytes (Hi-C data). **C** CRISPRi results in a human microglia cell line. We performed CRISPRi in a human microglial cell line (HMC3) expressing dCas9-ZIM3-KRAB using lentiviral delivery of three sgRNA guides targeting the rs74504435 region (G1, G2, and G4) and two non-targeting guides (NTC: mean of control guides). Bar plots show the mean *EGFR* relative expression compared to a no-guide control as assessed by qPCR; error bars are SEM; *N* = 3. Statistical analysis via one-way ANOVA followed by Tukey test, **p < 0.001; *p < 0.05.

We selected the second-highest-ranking V2G pair - rs74504435-*EGFR -* for further validation due to two main reasons. The LOAD risk allele at rs74504435 is associated with increased *EGFR* expression in multiple eQTL datasets (Fig. 5a), suggesting *EGFR* as a promising candidate for therapeutic targeting with existing *EGFR* inhibitors. This pair ranked highest in both the V2G_eQTL and V tiers, indicating robust support from diverse eQTL datasets and consistent functional annotations predicting a regulatory (enhancer) function for rs74504435 (Fig. 5a). Figure 5a visualizes this V2G pair using FILER tracks and Bellenguez GWAS summary statistics. rs74504435 regulates *EGFR* in 4 eQTLs: ROSMAP DLPFC, CommonMind DLPFC, GTEx Frontal Cortex, and GTEx cortex. It is also located within four brain-related enhancers from chromHMM and EpiMAP. We note that this top rs74504435 variant is also detected when using the most stringent pruning (LD = 0.1) and colocalization (PP.H4.abf >0.99) thresholds (Supplementary Data 4).

To investigate a potential regulatory role for rs74504435 on *EGFR* expression, we next validated this V2G pair by leveraging our collection of promoter-focused Capture C, ATAC-seq, and RNA-seq datasets from human brain-relevant cell types^48–50^, as well as Hi-C data from iPSC-derived astrocytes, microglia^51^, neurons, and oligodendrocytes^52^ (Supplementary Data 6). Via the ATAC-seq dataset, we observed that rs74504435 lies within open chromatin in several brain-relevant cell types, including iPSC-derived cortical neural progenitors and neurons^49^, primary astrocytes^50^, and iPSC-derived microglia^48^. We also observed a chromatin conformation capture contact (Capture C data) between this variant and *EGFR* in iPSC-derived cortical progenitors and neurons, primary astrocytes, and the microglial cell line HMC3. Using our RNA-seq datasets, we observed that *EGFR* was indeed expressed in these cell types^48–50^. From the Hi-C data, we observed that interactions between rs74504435 and *EGFR* exist in iPSC-derived neurons and oligodendrocytes. These findings are illustrated in Fig. 5b. Results for the other four top-ranked V2G pairs are summarized in Supplementary Data 7. While one of the three additional candidate SNPs (rs11585858) did show an open chromatin signature in iMg, the other 2 (rs4351 and rs2405442) resided in closed chromatin. Furthermore, none of the four V2G pairs was validated by Capture C in any of the cell types investigated; specifically, rs2405442 did not show any loop in any cell type, while rs11585858 had a loop to *CFAP126* in HMC3 cells and rs4351 had loops to *CYB561* in HMC3 and NPC, to *KCNH6* in NPC, and to *MARCHF10* in iMg. Therefore, we prioritized the V2G pair involving rs74504435 and *EGFR* for further experimental validation.

To validate the regulatory role for the region harboring rs74504435 and its influence on *EGFR* expression, we leveraged CRISPR interference (CRISPRi). We engineered the human microglial cell line HMC3 to stably express dCas9-KRAB (tagged with GFP). We transduced this line with lentivirus containing one of three sgRNAs targeting this region (G1, G2, and G4) or two control non-targeting sgRNAs (tagged with mCherry). After double selection for the presence of the guides and the dCas9-KRAB by FACS, we performed qPCR to assess *EGFR* levels. We found that two out of three targeting guides led to a consistent and significant decrease in *EGFR* expression levels compared to controls (one-way ANOVA *p* = 0.0002). G1 significantly decreased *EGFR* levels by 47% compared to the mean of the non-targeting controls (Tukey test *p* = 0.0004), and G2 by 27% (*p* = 0.04). G4 did not affect *EGFR* levels. Non-targeting guides did not show any effect when compared to a no-guide control. These results are shown in Fig. 5c.

Overall, our results support the hypothesis that the AD-associated variant rs74504435 is located within an enhancer region that regulates *EGFR* expression levels.

## Discussion

Functionally characterizing non-coding AD GWAS loci is crucial for successful drug target discovery, and FG datasets can aid in this challenging task. However, FG data are often sparse and unharmonized, which hinders progress in this field. Here, we leveraged hipFG^44^, a tool that integrates FG data into FILER, and selected brain-profiled FG data to systematically validate non-coding AD signals for their regulatory potential. Our new framework combines confluent FG evidence and directionality checks to prioritize V2G pairs for functional validation.

Starting with AD GWAS variants from Bellenguez et al.^5^, we defined independent loci using the 1000G^28^ LD structure. For each locus, we evaluated functional contexts, conducted Bayesian colocalization of GWAS and eQTL signals, and identified putative causal variants, genes, and V2G pairs. We performed in silico validation using independent assays and data sources, followed by experimental validation with promoter-focused Capture C, ATAC-seq, and CRISPRi. This approach identified five plausible V2G pairs with highest tiers: chr1:161186243:C:A_*NDUFS2* (tier 11), chr7:54881563:A:G_*EGFR* (tier 9), chr1:161186243:C:A_*FCER1G* (tier 9), chr7:100373690_*PVRIG* (tier 9), and chr17:63492371:G:A_*ACE* (tier 9). We successfully validated one of these pairs and its regulatory effect in a human microglia cell line.

Unlike prior post-GWAS methods, which rely on pre-selected top GWAS variants^11,25^, our framework utilized full GWAS summary statistics. We implemented QC steps to normalize genetic data against FG datasets in FILER, enabling a more systematic and comparable analysis of potential causal variants, genes, and V2G pairs. Our tiering system, which integrates support from eQTL, enhancer–promoter interactions, variant effects, and gene/protein expression, enabled improved prioritization of V2G pairs.

By leveraging full GWAS summary statistics and LD expansion in post-GWAS analyses, we uncovered additional candidate causal signals that were missed in previous studies^5^. When we compared the number of colocalization signals (the first step in our pipeline, GTEx brain data alone) to analyses that did not perform LD pruning or expansion^11^, we found that using the full summary statistics, rather than only top variants, yielded 4.5 times more candidate variants (9144 vs. 2024), 5 times more unique colocalized variants (2040 vs. 408), and twice as many candidate target genes (1529 vs. 762) across all GTEx data. Details for the candidate variants, colocalized variants, and corresponding targets from both the top variant analyses as well as the genome-wide approach we used in our current analyses can be found in Supplementary Data 8-11.

In our multi-tiered framework, colocalization served as an initial, relatively permissive filter rather than the final criterion. We then applied additional validation steps and external datasets to refine these signals. While others have used more stringent cutoffs as final criteria, we selected a >0.7 threshold at the first stage to capture a broader set of candidate variant-gene pairs (6971 at >0.7 vs 3404 at >0.9; Supplementary Data 3, 9). Importantly, over half of the signals confirmed in our in silico validation (23/41) would have been missed had we used stricter thresholds (>0.95 or >0.99) at the outset.

Using eQTL data for colocalization explained only a small fraction of the GWAS signals^53^. In our approach, we did not restrict analysis to signals with EPI support. Instead, we first prioritized eQTL colocalization and then sought additional in silico validation. 41 eQTLs were validated using independent eQTL datasets, and 37 out of 41 also had EPI support. EPI data can help prioritize V2G pairs when eQTL signals are weaker (e.g., loci chr7:100374211:A:G_CNPY4 and chr7:100373690:T:C_AP4M1). However, V2G pairs supported by both types of evidence are not always superior, as discrepancies may arise from biological differences or the uneven availability of FG assays, which limits cross-cell-type comparisons. More cell-type-specific assays, currently unavailable, could further improve V2G pair identification.

A potential concern when integrating multiple QTL datasets is the risk of circularity, where validation may inadvertently rely on overlapping information. In our study, we minimized this risk by strictly separating discovery from validation. Candidate loci were defined exclusively using GTEx v8^29^ eQTL data, and validation was performed using independent datasets (MetaBrain^39^, MiGA^42^, and the eQTL Catalogue^45^), which are based on distinct cohorts, tissue sources, and analytical pipelines. Because these datasets do not share individuals or genotype data with GTEx, concordant colocalization signals represent independent replication rather than re-analysis of the same association. Importantly, we also incorporated orthogonal functional data types, including chromatin interaction datasets (3DGenome^41^, 4DGenome^40^, Nott et al.^43^), enhancer annotations (Nott et al.^43^), and TFBS information^27^, which are methodologically distinct from QTL-based approaches. This multi-tiered framework reduces the likelihood of circularity and increases the robustness of our gene prioritization strategy.

Using CRISPRi in a microglial cellular setting, we successfully validated *EGFR* as a target gene whose regulation is influenced by the AD variant rs74504435. The *EGFR* (Epidermal Growth Factor Receptor) gene product is a receptor tyrosine kinase that controls cell proliferation, survival, differentiation, and inflammation. In AD, it has been connected to disease progression^54^, with elevated levels associated with increased Aβ plaque formation. Additionally, *EGFR* inhibition modulates neuroinflammation and cognitive function in AD animal models^55^.

Interestingly, we found evidence supporting a role for the rs74504435-*EGFR* V2G pair in multiple brain-relevant cell types: (1) rs74504435 resides in open chromatin regions of neurons, microglia, and astrocytes in ATAC-seq data; (2) rs74504435 contacts the 3′ UTR region of *EGFR* in promoter-focused Capture C data generated from microglia, neurons and astrocytes; (3) rs74504435 also contacts the *EGFR* promoter in Hi-C data derived from iPSC-derived oligodendrocytes and neurons; (4) rs74504435 is associated with *EGFR* expression in single-nucleus RNA-seq data from astrocytes (*p* = 3.2 × 10^−^^24^, Z-score = −12.53) and oligodendrocyte progenitor cells (*p* = 0.003, Z-score = −2.95) derived from the dorsolateral prefrontal cortex in ROSMAP samples (Supplementary Fig. 4)^56^. In Bellenguez et al., another variant, rs76928645, was reported to interact with *EGFR*. Using our promoter-focused Capture C data and ATAC-seq data, we observed a loop via the Capture C experiment in NPC cells, but the loop does not reside in the open chromatin region. As a consequence, we conclude that *EGFR*’s functional role in AD likely involves several cell types, and further research is required to investigate whether the underlying mechanisms are distinct or shared across different brain cell types and whether pathogenesis is driven by a specific cell type.

Because APOE has an outsized effect on AD genetics and a complex haplotype structure, we removed this locus and the entire chr19 in the main analyses. To assess its contribution, we compared colocalization signals with and without chr19 (Supplementary Data 10). chr19 contributed 15% of candidate variants (1355/9144), covering 40% of candidate regions (417/1043), and accounted for 26% of colocalized signals. These results confirm the APOE region as a major driver of colocalization but also highlight substantial signals outside APOE and other chr19 loci, indicating additional genes and pathways likely contribute to AD pathogenesis.

There are two limitations to our study that can be addressed in future research. First, we restricted our analyses to SNPs. Indels were excluded because they are not consistently represented in most QTL datasets currently available, which limited our ability to integrate them across resources. Additionally, out of the three QTL data sources used for validation (eQTL Catalogue^45^, MetaBrain^39^, and MiGA^42^), only eQTL Catalogue had indels. Furthermore, indel detection and quantification vary substantially across genotyping arrays, in contrast to SNPs, which are more uniformly assayed. As QTL studies increasingly leverage whole-genome sequencing and standardized approaches for indel calling, incorporating indels into future pipelines will be important, especially given evidence for their role in LOAD (e.g., the *TOMM40* region^57^). Second, our analyses were performed on GWAS data from European-ancestry populations. We did not include non-European GWAS in these analyses because of their small sample sizes. Our modular framework could, in principle, be adapted to diverse cohorts by incorporating ancestry-matched LD reference panels (e.g., 1000 Genomes Project^28^, Alzheimer’s Disease Sequencing Project^58^) and population-specific QTL datasets. The current scarcity of functional genomics resources from non-European populations is a major barrier. As more multi-ancestry GWAS and QTL datasets become available, extending this framework to diverse cohorts will be essential for improving the generalizability of genetic discoveries and for reducing health disparities in AD.

In conclusion, by combining an unbiased, confluent context identification framework, in silico V2G pair validation using QTL directionality, and a comprehensive scoring system that considers variant, gene, and V2G pair effects, we identified 41 AD-associated V2G pairs. The FILER-curated brain FG datasets and hipFG-harmonized FG data were instrumental to this success. Among the top findings, five V2G pairs achieved tier 9 or higher, and we demonstrated that AD-associated variant rs74504435 (chr7:54881563:A:G) resides in a regulatory region influencing *EGFR* expression, as validated by promoter-focused Capture C and CRISPRi functional experiments. Given that *EGFR* inhibitors are already approved for cancer therapy, *EGFR* could represent a promising candidate for repurposing as a therapeutic target for LOAD. Our framework and results provide valuable insights for future AD research.

## Methods

### Description of the AD GWAS

We analyzed Stage 1 genome-wide summary stats from Bellenguez et al.^5^, (downloaded from GWAS catalog http://ftp.ebi.ac.uk/pub/databases/gwas/summary_statistics/GCST90027001-GCST90028000/GCST90027158/). This dataset aggregates samples from International Genomics of Alzheimer’s Project (IGAP), with the inclusion of cohorts from European Alzheimer’s & Dementia BioBank (EADB), Alzheimer’s Disease Genetics Consortium (ADGC), and others, including clinically defined AD cases/controls and UK Biobank dementia samples, totaling 788,989 individuals with an effective sample size of 382,472. It contains 19,767,628 SNPs and 1,333,486 indels; our analysis focused on SNPs. For details, refer to the original publication^5^.

### Identifying regions of interest for downstream analyses

We performed LD pruning using the 1000 Genomes Phase 3 EUR reference panel on all genome-wide significant variants (*p* < 5 × 10^−^^8^), identifying pairwise-independent tag variants (*r*^2^ < 0.7) using a 500 kb window. For each tag variant, we defined an analysis region by including variants that are in LD with the tag variant (*r*^2^ > =0.7), are within 1 M base pairs (bps), and are within 1000 variants of the tag. These variants, along with the tag variants, are considered candidate regulatory variants. Each analysis region is bounded by the outermost variants in LD with the tag. We note that not only the candidate variants, but all variants within these regions, even if some have no association with AD, will be included in colocalization analyses.

The LD-based thresholds used in our analyses are adjustable. Here, we use an *r*^2^ < 0.7 threshold for pruning the set of genome-wide significant (*p* < 5 × 10^−^^8^) variants. This threshold effectively removes variants in high LD (*r* > 0.83) and allows for ensuring the relative pairwise independence between the variants in the pruned set. At the LD expansion step, using *r*^2^ > 0.7 adds all the other variants that are in high LD (*r* = 0.83 or more) with the tag variants from the pruned set. Similar LD thresholds were used in previous studies (e.g., non-coding variant analysis^59^; or selecting a maximally informative set of SNPs^60^).

### Genome partition analysis of all candidate regulatory variants

Variants were categorized into different genomic categories using the UCSC knownGene^61,62^, UCSC RepeatMasker^62,63^, and GENCODE v43 lncRNA annotations^64^ for the GRCh38/hg38 genome build. The 5′ UTR exons and introns, exons, introns, and 3′ UTR exons and introns were extracted from the knownGene annotation for each protein-coding gene. Promoter annotations were defined as 1000 bps genomic regions upstream of the transcription start site. To create a hierarchical genomic partition into disjoint 5′ UTR exon, 5′ UTR intron, 3′ UTR exon, 3′ UTR intron, promoter, exonic, and intronic regions (in this order), each region set was obtained by subtraction of the merged regions higher in the genomic hierarchy. For example, starting from merged 5′ UTR exonic regions, distinct 5′ UTR intronic regions were obtained by subtraction of 5′ UTR exonic regions from the merged 5′ UTR intronic regions, and 3′ UTR exon regions were obtained by subtracting both 5′ UTR exonic and intronic regions.

During analysis, GWAS variants were then assigned to mutually exclusive genomic element annotations using the created hierarchy: 5′ UTR exon > 5′ UTR intron > 3′ UTR exon > 3′ UTR intron > promoter > mRNA exon > mRNA intron. Overlaps, if any, with repeat element annotations (e.g., SINE, LINE) were also reported for all variants. Variants overlapping any of GENCODE lncRNA annotations were additionally classified into lncRNA exonic and/or lncRNA intronic variants. A variant not overlapping with any class of elements above (mRNA, lncRNA, repeat) was classified as intergenic.

### Functional genomic annotations of all candidate regulatory variants

Genomic annotations from the FILER (functional genomic database which contains harmonized genomic annotation data across >30 primary data sources^26^) were used. 140 of these are used in the “Characterization”, i.e., discovery phase (including genome partition analyses, colocalization analyses, and unbiased confluent context identification), while 34 are used in “In silico validation”. See Supplementary Data 11 for details. In the discovery phase, the datasets included fundamental genome annotations and reference variant information (dbSNP^65^, GENCODE gene annotations^64^), genome-wide HOMER^27^ transcription factor binding tracks, and 140 brain-related FILER tracks (tissues and cells only) for variant characterization, including enhancers (10 from ROADMAP^30^, 55 from EpiMAP^31^), QTLs (26 from GTEx v8^29^), and epigenetics (49 from ENCODE^66^).

### Variant-Gene (V2G) pair identification (colocalization)

We hypothesized that putative causal variants affect gene expression in a cis-regulatory manner. To do so, we have performed a colocalization analysis within each genomic region to identify a shared (AD+eQTL) causal variant, if any. The colocalization analysis considers all possible causal configurations when computing the posteriors for colocalization (H4), including a null (H0) probability of no association for both AD and eQTL in the region, and the probabilities for AD-only association (H1), eQTL-only association (H2), and different/non-colocalized causal variants (H3). P(H4) provides an accurate measure of the colocalization probability, with the FDR corresponding to the cumulative probability of the alternative scenarios, P(H0) + P(H1) + P(H2) + P(H3) = 1 − P(H4), i.e., the probability that the AD and eQTL do not share the same causal variant in the region. A PP.H4.abf value of >0.95 is equivalent to FDR < 5%.

By aligning GWAS and eQTL signals via colocalization, we can identify genes most likely affected by disease-associated variants. Using the COLOC R package v5.2.3^67^, we performed Bayesian colocalization on 9144 candidate variants against nominally significant eQTLs from 13 brain tissues in the GTEx v8 dataset. We defined a V2G pair as colocalized if the candidate variant was the most likely causal variant in the locus (SNP.H4.abf >0.5), the posterior probability for colocalization was greater than 0.7 (PP.H4.abf), and the locus contained more than one variant.

### Transcription factor binding site (TFBS) disruption analyses of all candidate regulatory variants

HOMER (Hypergeometric Optimization of Motif EnRichment)^27^ is a custom motif database derived from high-quality ChIP-Seq data. A positional weight matrix (PWM) represents transcription factor (TF) DNA binding specificities. The delta PWM score (difference between reference and alternate alleles) estimates binding activity changes due to nucleotide variation. A candidate causal variant is selected for the next steps if it disrupts a TF binding site with a delta PWM score >|2| for any TF.

### Enhancers overlap analyses of all candidate regulatory variants

Enhancers are DNA regulatory elements that activate gene transcription by forming chromatin loops to interact with target genes in a cell-type-specific manner. Databases like ROADMAP^30^ and EpiMAP^31^ catalog enhancers across various cell types and tissues. A candidate causal variant is considered potentially regulatory if it overlaps a brain enhancer found in either ROADMAP^30^ or EpiMAP^31^.

### Unbiased confluent context identification of a putative causal V2G

We define a putative causal V2G pair as one with strong colocalization (“Variant-Gene (V2G) pair identification (colocalization)” section). The associated genes are considered putative causal genes. The putative causal variant is predicted to disrupt a TFBS (“Transcription factor binding site (TFBS) disruption analyses of all candidate regulatory variants” section) and overlap a tissue- or cell-type-specific enhancer (“Enhancers overlap analyses of all candidate regulatory variants” section) in any brain FG data. To identify which AD genetic signals may function in the brain-specific confluent context, we included all relevant tracks in FILER. We then required the colocalization tissue context to match the enhancers’, forming the final set of putative causal V2G pairs. A pair is excluded if it fails to meet any of these criteria.

### Harmonizing in silico datasets by hipFG

In the validation phase, selected brain tracks include chromatin interactions (5 from 3DGenome^41^, 1 from 4DGenome^40^, 2 from Nott et al.^43^), QTLs (18 from eQTL Catalogue^45^, 4 from MetaBrain^39^, 4 from MiGA^42^). These tracks were generated from primary tissues/cell types but not cell lines. Each dataset was processed using hipFG (Harmonization and Integration Pipeline for Functional Genomics)^44^, an automated pipeline that standardizes, indexes, and integrates diverse functional genomics data (e.g., EPI, genomic intervals, QTLs) for scalable, searchable analysis.

### In silico validation on candidate variant-gene pairs and genes

We validate selected V2G pairs and genes in silico using a set of independent FG resources (Fig. 1, “In silico validation”). Validation requires evidence from at least one, but not all, of the following categories. For V2G pairs, topologically associating domain (TAD) validation was based on TADs shared by the 3DGenome^41^, where a V2G pair was considered in silico validated if it overlapped with both anchors of the interaction. Enhancer–promoter interaction (EPI) validation included data profiled by PLAC-seq, 3C, 4C-Seq, 5C, Hi-C, ChIA-PET, and promoter-focused Capture C from Nott et al.^43^, 3DGenome^41^, and 4DGenome^40^ using only brain-related datasets; a V2G pair was in silico validated if it overlapped with both anchors of the interaction. Bulk tissue eQTL validation used brain tissue or region-related eQTLs from the eQTL Catalogue^45^ and the MetaBrain^39^, covering four brain regions. A V2G pair was in silico validated if it overlapped with, contained the same effect allele, and carried the same effect direction on the same gene as any eQTL profiled in these resources.

For genes, validation was based on two sources of evidence. Biological context was derived from the Human Protein Atlas (HPA)^68,69^; a gene was in silico validated if it was identified in the HPA as a protein-coding gene expressed in any brain region or cell type. Disease specificity was assessed using information from the Agora AMP-AD platform^37^, where a gene was in silico validated if it was included in the nominated list of genes.

### Independent regulatory evidence and potential functions of selected variants

In addition to functional evidence from in silico validation, annotations from other sources can further support a variant’s regulatory or functional potential. As with previous analyses, evidence from any (not all) of the following categories is sufficient for confirming a variant as in silico functional.

Functional annotation based on active histone marks (H3K27ac) was derived from ENCODE^66^, where a variant was considered functional if it significantly overlapped any active histone mark peak (*q*-value < 5%). Open chromatin regions were analyzed using ATAC-seq data from ENCODE^66^, and a variant was considered functional if it was located within an ATAC-seq peak. Variant effect prediction was assessed using the Combined Annotation Dependent Depletion (CADD) score^70^ and RegulomeDB2 score^71^. CADD scores estimate variant deleteriousness, with higher scores indicating greater impact, while RegulomeDB2 integrates functional genomic assays to assign heuristic rankings for regulatory potential. A variant was considered functional if it had a CADD score >10 or a RegulomeDB2 score of 1a-1e. Genetic association evidence was evaluated using the GWAS Catalog^72^ and NIAGADS GenomicsDB^73^ to determine whether a putative causal variant was linked to Alzheimer’s disease (AD)-related traits. The GWAS Catalog contains variant-trait associations from over 130,000 GWASs across more than 18,000 traits (as of February 2025), and NIAGADS GenomicsDB is an interactive AD genetics database with 476.9K annotated variants from over 80 AD GWASs. A variant was considered functional if, in addition to being reported in Bellenguez et al.^5^ AD GWAS, it was also associated with a phenotype in any other AD GWAS.

### Ranking of variant-gene (V2G) pairs (tier system)

Given the large number of variant-gene pairs identified after the confluence analyses, it remains challenging for wet-lab scientists to prioritize pairs for further functional work. We introduced a tiered system that integrates evidence from eQTLs (V2G_eQTL tier), EPIs (V2G_EPI tier), variants (V_tier), and genes (G_tier), each combining data from multiple sources (*Methods: Ranking of variant-gene pairs (Tier system)*).

The V2G_eQTL tier assessed four features: directionality across non-GTEx eQTLs (Metabrain^39^, eQTL Catalogue^45^), the presence of non-GTEx brain eQTLs, consistency between non-GTEx and GTEx brain-region eQTLs, and a negative, consistent Z-score across brain regions (we note that our directionality/Z-score consistency checks are not using formal hypothesis testing). Each criterion was assigned a score of 1, and the total V2G_eQTL tier score ranged from 1 to 4.

The V2G_EPI tier evaluated whether a V2G pair appeared in brain enhancer–promoter interaction datasets from 3DGenome^41^, 4DGenome^40^, or Nott et al.^43^. A score of 1 was assigned for each presence, and the total V2G_EPI tier ranged from 0 to 2.

The V_tier evaluates variant-level properties, including overlap with active histone marks, open chromatin regions, CADD scores^70^, RegulomeDB2 rankings^71^, and statistical significance in GWAS (GWAS Catalog^72^, NIAGADS GenomicsDB^73^). Variants meeting the ‘functional’ definition in each category were assigned a score of 1, resulting in a total V_tier range of 0–4.

The G_tier considered gene-level evidence, including nomination by the AMP-AD Agora^37^ and expression in brain regions or cell types according to the Human Protein Atlas (HPA)^68,69^. A score of 1 was assigned for each presence, and the total G_tier ranged from 0 to 2.

The overall tier for the V2G pair is the sum of all four components. The overall tier ranges from 1 to 11.

### Hi-C data generation from iPSC cells for functional validation

Details of the iPSC cells used in this analysis can be found in the previous two studies^51,74^. To understand the potential function of the intergenic SNP rs74504435, we examined chromatin interactions using Hi-C analysis in iPSC-derived astrocytes, microglia cells, oligodendrocytes, and neurons^52^. An in situ Hi-C library was prepared using the protocol adapted from Rao et al.^75^. For each library, 450~550 million paired-end reads at 150 bps length were obtained. Sequencing data were processed using BWA^76^ to map each read end separately to the GRCh38 reference genomes. Duplicate and non-uniquely mapped reads were removed. For each library, over 270 million non-redundant, uniquely mapped, paired reads were used for further analysis. For robust enhancer–promoter interaction mapping, Chromatin loops were called using HiCorr^77^ to correct bias and LoopEnhance^78^ to remove noise.

### Chromatin interaction analysis

We queried our existing genomic datasets (see Supplementary Data 12), including high-resolution promoter-focused Capture C, ATAC-seq, and RNA-seq from brain-relevant cell types (iPSC-derived neural progenitors and neurons^48^, iPSC-derived microglia and the human microglia cell line HMC3^49^, and primary astrocytes^50^) to assess whether candidate variants were residing in open chromatin regions and contacting the promoter of an expressed gene.

Chromatin interactions calls obtained from promoter-focused Capture C datasets were available from the references provided for each cell type: briefly, paired-end reads were pre-processed using the HiCUP pipeline^79^ and aligned with bowtie2^80^ to the reference genome, and significant interactions at 1-DpnII and 4-DpnII fragment resolutions were called using CHiCAGO^80^ with default parameters except for binsize, which was set to 2500. Open chromatin region calls from ATAC-seq datasets were also available from the same referenced studies: peaks were called using the ENCODE ATAC-seq pipeline (https://www.encodeproject.org/atac-seq/), selecting the resulting IDR optimal peaks. When more than two technical replicates were available for one cell type (i.e., for primary astrocytes, iPSC-derived microglia, and HMC3 cells), peaks were also called using a custom pipeline (“reproducible peaks”), where a peak is called if it’s present in the majority of the technical replicates available, and the union of IDR optimal peaks and reproducible peaks was used in the analysis. The queries to intersect these annotations with the candidate variants of interest were performed in GRCh37/hg19, and the results were lifted over to GRCh38 with the UCSC tool liftOver^80^ for comparison with other annotations.

### EGFR sgRNA design

The genomic coordinate location for rs74504435 was obtained using the UCSC genome browser (build GRCh37/hg19). This genomic coordinate for rs74504435 plus and minus 200 bps was then entered into the software CHOPCHOP^81^ to generate a table of possible single guide RNAs (sgRNAs) for CRISPRi (repression) using Cas9. The Cas-OffFinder software^82^ was used to access the off-target mismatches of the possible sgRNAs generated from CHOPCHOP. After assessing the efficiency and off-target mismatches, three sgRNAs targeting rs7450443 (G1, G2, G4) were selected. Two non-targeting control guides (N2 and NTC3, Millipore Sigma) were used as negative controls for the CRISPRi experiments. As a positive control, we utilized a guide targeting an enhancer of the gene *TSPAN14*, since we previously validated this construct for CRISPRi experiments in HMC3.

### Cloning the sgRNAs in a lentiviral plasmid

We leveraged a lentiviral vector created in the Chesi lab (SL33 Lenti-sgRNA(Tp2)-mCherry) to generate the backbone and the insert required for NEB HiFi DNA Assembly cloning. This lentiviral vector contains a U6 promoter driving the expression of one sgRNA; it also contains an sgRNA scaffold region and mCherry as a selection marker. To clone the sgRNA of interest into this vector, forward and reverse primers were designed and ordered through Azenta. The forward primer included: the sequence of the sgRNA of interest, complementary bases to the sgRNA scaffold, and overhanging bases (complementary to the vector - which is needed for Hifi cloning). The reverse primer was designed in such a way that when used with the forward primer in PCR, the amplicon would be the HiFi insert, i.e., the sequence of the sgRNA of interest, the sgRNA scaffold, and overhanging bases complementary to parts of the HiFi backbone. The SL33 Lenti-sgRNA(Tp2)-mCherry vector was digested with restriction enzymes XhoI and BsrG1 and run on a gel to isolate and extract the HiFi backbone. The forward and reverse primers were used in a PCR cycle with the SL33 Lenti-sgRNA(Tp2)-mCherry as template in order to obtain the HiFi insert. The backbone and the insert were combined in HiFi cloning to generate the respective sgRNA lentiviral plasmids. The plasmids were then transformed using NEB 5 alpha competent bacteria. After colony picking and miniprep, plasmids were submitted to Plasmidsaurus for Nanopore sequencing for validation. We cloned three sgRNAs designed to target rs74504435 (G1, G2, G4) and two non-targeting sgRNAs (N2 and NTC3).

### Virus generation

On day 0, 400,000 HEK 293T cells per well were plated on PDL-coated 6-well plates. On day 1, cells were transfected with the respective sgRNA lentiviral plasmid in addition to envelope (Addgene plasmid #12259) and packaging (Addgene plasmid #12260) plasmids using the Lipofectamine 3000 reagent. On day 2, complete media changes were performed. On day 4, media from the HEK Cells (Virus Day Two) were collected, filtered through a 0.45-micron filter, aliquoted, and frozen/stored in the −80 °C.

### Transducing HMC3 helper line cells

We generated a CRISPRi helper line by transducing the human microglial line HMC3 (ATCC #CRL-3304) with a lentiviral vector encoding Zim3 Krab dCas9 and GFP as a selection marker (Addgene plasmid #188778). This line (HMC3-zim3) was plated in a 6-well format - 200,000 cells per well on day 0. On day 1, cells were transduced with Day Two virus, and polybrene was used to aid in transduction efficiency. Due to the toxic nature of polybrene, if cells are exposed to it for too long, a complete media change was performed 18 h later (day 2). On the same day, cells were later moved from the 6-well format to a 100 mm plate in order to expand them for fluorescence-activated cell sorting (FACS). After cells reached about 80–100% confluency on the 100 mm plate, FACS was performed on these transduced cells as well as naïve HMC3 cells (used as a baseline for fluorescence) at the Flow Cytometry Core Laboratory at CHOP. Double positive cells were selected, i.e., the top 50% of cells that had both GFP fluorescence (indicating that these cells have dCas9) and mCherry fluorescence (indicating that these cells had the sgRNA lentiviral vector) were chosen. After recovering from flow sorting, cells were cultured and expanded until there were enough to perform qPCR experiments.

### qPCR experiment

qPCR primers for *EGFR* (spanning exons 7–9), *TSPAN14* (spanning exons 8–9), and *GAPDH* (spanning exons 2–3) were ordered through IDT. After cells were expanded post-FACS, cells were plated in a 6-well format (220,000 cells per well). 24 h after plating, cells were pelleted, and RNA extraction was immediately performed using the Qiagen RNeasy Plus micro kit and QIAShredder kit. Using Applied Biosystems’ High-Capacity cDNA reverse transcription kit, cDNA synthesis was also performed on the same day as RNA extraction. Power SYBRgreen PCR master mix from Applied Biosystems was used to perform standard comparative qPCR with primers for the two target and one housekeeping gene (*EGFR*, *TSPAN14*, and *GAPDH*). Three biological replicates in total were performed for each condition.

### Extra information on the sgRNAs

Sequences of sgRNAs targeting rs7450443 w/o PAM sequence:

G1: 5′ TAGGCCTGAATGTCAATCAC 3′

G2: 5′ AGTGTGTTGAGTGTGAACAC 3′

G4: 5′ GTGTCAGCTCTCACTGAAAG 3′

Sequences of non-targeting guides

N2: 5′ CGCTTCCGCGGCCCGTTCAA 3′ was ordered from Millipore Sigma and was delivered in the form of a virus

NTC3: 5′ CCCGAGCAGTGGCTCGCTA 3′ is a non-targeting guide ordered from Millipore Sigma

Sequence of positive control - sgRNA targeted to the enhancer of *TSPAN14* (Tspan14_enh): 5′ CTTAGGCGCTGCATACCGTA 3′

## Supplementary information

Supplementary information

Supplementary Data 1

Supplementary Data 2

Supplementary Data 3

Supplementary Data 4

Supplementary Data 5

Supplementary Data 6

Supplementary Data 7

Supplementary Data 8

Supplementary Data 9

Supplementary Data 10

Supplementary Data 11

Supplementary Data 12

## Data Availability

The analyzed AD GWAS summary statistics data is available in the GWAS catalog ([https://ftp.ebi.ac.uk/pub/databases/gwas/summary_statistics/GCST90027001-GCST90028000/GCST90027158/]). The selected FG datasets used in this study are available from the FILER database and in Supplementary Data 12.

## Code availability

Reported analyses were performed using the adapted INFERNO pipeline (https://bitbucket.org/wanglab-upenn/bash-INFERNO).

## References

[1] Gatz, M. et al. Heritability for Alzheimer’s disease: the study of dementia in Swedish twins. *J. Gerontol. A Biol. Sci. Med. Sci.* **52**, M117–M125 (1997). 9060980 10.1093/gerona/52a.2.m117

[2] Corder, E. H. et al. Gene dose of apolipoprotein E type 4 allele and the risk of Alzheimer’s disease in late onset families. *Science* **261**, 921–923 (1993). 8346443 10.1126/science.8346443

[3] Escott-Price, V. et al. Common polygenic variation enhances risk prediction for Alzheimer’s disease. *Brain* **138**, 3673–3684 (2015). 26490334 10.1093/brain/awv268 PMC5006219

[4] Lambert, J. C. et al. Meta-analysis of 74,046 individuals identifies 11 new susceptibility loci for Alzheimer’s disease. *Nat. Genet.* **45**, 1452–1458 (2013). 24162737 10.1038/ng.2802 PMC3896259

[5] Bellenguez, C. et al. New insights into the genetic etiology of Alzheimer’s disease and related dementias. *Nat. Genet.* **54**, 412–436 (2022). 35379992 10.1038/s41588-022-01024-z PMC9005347

[6] Wightman, D. P. et al. A genome-wide association study with 1,126,563 individuals identifies new risk loci for Alzheimer’s disease. *Nat. Genet.* **53**, 1276–1282 (2021). 34493870 10.1038/s41588-021-00921-z PMC10243600

[7] Heneka, M. T. et al. Neuroinflammation in Alzheimer’s disease. *Lancet Neurol.* **14**, 388–405 (2015). 25792098 10.1016/S1474-4422(15)70016-5 PMC5909703

[8] Heppner, F. L., Ransohoff, R. M. & Becher, B. Immune attack: the role of inflammation in Alzheimer disease. *Nat. Rev. Neurosci.* **16**, 358–372 (2015). 25991443 10.1038/nrn3880

[9] Sims, R. et al. Rare coding variants in PLCG2, ABI3, and TREM2 implicate microglial-mediated innate immunity in Alzheimer’s disease. *Nat. Genet.* **49**, 1373–1384 (2017). 28714976 10.1038/ng.3916 PMC5669039

[10] Novikova, G. et al. Integration of Alzheimer’s disease genetics and myeloid genomics identifies disease risk regulatory elements and genes. *Nat. Commun.* **12**, 1610 (2021). 33712570 10.1038/s41467-021-21823-y PMC7955030

[11] Amlie-Wolf, A. et al. Inferring the molecular mechanisms of noncoding Alzheimer’s disease-associated genetic variants. *J. Alzheimers Dis.* **72**, 301–318 (2019). 31561366 10.3233/JAD-190568 PMC7316086

[12] Bulger, M. & Groudine, M. Functional and mechanistic diversity of distal transcription enhancers. *Cell* **144**, 327–339 (2011). 21295696 10.1016/j.cell.2011.01.024 PMC3742076

[13] Wittkopp, P. J. & Kalay, G. Cis-regulatory elements: molecular mechanisms and evolutionary processes underlying divergence. *Nat. Rev. Genet.* **13**, 59–69 (2011). 22143240 10.1038/nrg3095

[14] Ong, C. T. & Corces, V. G. Enhancer function: new insights into the regulation of tissue-specific gene expression. *Nat. Rev. Genet.* **12**, 283–293 (2011). 21358745 10.1038/nrg2957 PMC3175006

[15] Corradin, O. & Scacheri, P. C. Enhancer variants: evaluating functions in common disease. *Genome Med.* **6**, 85 (2014). 25473424 10.1186/s13073-014-0085-3 PMC4254432

[16] Schwartzentruber, J. et al. Genome-wide meta-analysis, fine-mapping and integrative prioritization implicate new Alzheimer’s disease risk genes. *Nat. Genet.* **53**, 392–402 (2021). 33589840 10.1038/s41588-020-00776-w PMC7610386

[17] Bryois, J. et al. Cell-type-specific cis-eQTLs in eight human brain cell types identify novel risk genes for psychiatric and neurological disorders. *Nat. Neurosci.* **25**, 1104–1112 (2022). 35915177 10.1038/s41593-022-01128-z

[18] Kosoy, R. et al. Genetics of the human microglia regulome refines Alzheimer’s disease risk loci. *Nat. Genet.* **54**, 1145–1154 (2022). 35931864 10.1038/s41588-022-01149-1 PMC9388367

[19] King, E. A., Davis, J. W. & Degner, J. F. Are drug targets with genetic support twice as likely to be approved? Revised estimates of the impact of genetic support for drug mechanisms on the probability of drug approval. *PLoS Genet.* **15**, e1008489 (2019). 31830040 10.1371/journal.pgen.1008489 PMC6907751

[20] Razuvayevskaya, O., Lopez, I., Dunham, I. & Ochoa, D. Genetic factors associated with reasons for clinical trial stoppage. *Nat. Genet.* **56**, 1862–1867 (2024). 39075208 10.1038/s41588-024-01854-z PMC11387188

[21] Minikel, E. V., Painter, J. L., Dong, C. C. & Nelson, M. R. Refining the impact of genetic evidence on clinical success. *Nature* **629**, 624–629 (2024). 38632401 10.1038/s41586-024-07316-0 PMC11096124

[22] Schilder, B. M. & Raj, T. Fine-mapping of Parkinson’s disease susceptibility loci identifies putative causal variants. *Hum. Mol. Genet.* **31**, 888–900 (2022). 34617105 10.1093/hmg/ddab294 PMC8947317

[23] Li, M. et al. Integrative functional genomic analysis of human brain development and neuropsychiatric risks. *Science* **362**, eaat7615 (2018). 30545854 10.1126/science.aat7615 PMC6413317

[24] Chen, Z. et al. Functional genomics provide key insights to improve the diagnostic yield of hereditary ataxia. *Brain* **146**, 2869–2884 (2023). 36624280 10.1093/brain/awad009 PMC10316781

[25] Kuksa, P. P. et al. SparkINFERNO: a scalable high-throughput pipeline for inferring molecular mechanisms of non-coding genetic variants. *Bioinformatics* **36**, 3879–3881 (2020). 32330239 10.1093/bioinformatics/btaa246 PMC7320617

[26] Kuksa, P. P. et al. FILER: a framework for harmonizing and querying large-scale functional genomics knowledge. *NAR Genom. Bioinform.* **4**, lqab123 (2022). 35047815 10.1093/nargab/lqab123 PMC8759563

[27] Heinz, S. et al. Simple combinations of lineage-determining transcription factors prime cis-regulatory elements required for macrophage and B cell identities. *Mol. Cell* **38**, 576–589 (2010). 20513432 10.1016/j.molcel.2010.05.004 PMC2898526

[28] Genomes Project, C. et al. A global reference for human genetic variation. *Nature* **526**, 68–74 (2015). 26432245 10.1038/nature15393 PMC4750478

[29] Consortium, G. T. The GTEx Consortium atlas of genetic regulatory effects across human tissues. *Science* **369**, 1318–1330 (2020). 32913098 10.1126/science.aaz1776 PMC7737656

[30] Roadmap Epigenomics, C. et al. Integrative analysis of 111 reference human epigenomes. *Nature* **518**, 317–330 (2015). 25693563 10.1038/nature14248 PMC4530010

[31] Hoon, D. S. B., Rahimzadeh, N. & Bustos, M. A. EpiMap: Fine-tuning integrative epigenomics maps to understand complex human regulatory genomic circuitry. *Signal Transduct. Target. Ther.* **6**, 179 (2021). 33966052 10.1038/s41392-021-00620-5 PMC8106674

[32] Scoville, D. W., Kang, H. S. & Jetten, A. M. GLIS1-3: emerging roles in reprogramming, stem and progenitor cell differentiation and maintenance. *Stem Cell Investig.* **4**, 80 (2017). 29057252 10.21037/sci.2017.09.01 PMC5639011

[33] Bu, S., Lv, Y., Liu, Y., Qiao, S. & Wang, H. Zinc finger proteins in neuro-related diseases progression. *Front. Neurosci.* **15**, 760567 (2021). 34867169 10.3389/fnins.2021.760567 PMC8637543

[34] Calderari, S. et al. Molecular genetics of the transcription factor GLIS3 identifies its dual function in beta cells and neurons. *Genomics* **110**, 98–111 (2018). 28911974 10.1016/j.ygeno.2017.09.001

[35] von Bernhardi, R., Cornejo, F., Parada, G. E. & Eugenin, J. Role of TGFbeta signaling in the pathogenesis of Alzheimer’s disease. *Front. Cell Neurosci.* **9**, 426 (2015). 26578886 10.3389/fncel.2015.00426 PMC4623426

[36] Town, T. et al. Blocking TGF-beta-Smad2/3 innate immune signaling mitigates Alzheimer-like pathology. *Nat. Med.* **14**, 681–687 (2008). 18516051 10.1038/nm1781 PMC2649699

[37] AMP-AD AGORA.

[38] Kerimov, N. et al. eQTL Catalogue 2023: new datasets, X chromosome QTLs, and improved detection and visualisation of transcript-level QTLs. *PLoS Genet.* **19**, e1010932 (2023). 37721944 10.1371/journal.pgen.1010932 PMC10538656

[39] de Klein, N. et al. Brain expression quantitative trait locus and network analyses reveal downstream effects and putative drivers for brain-related diseases. *Nat. Genet.* **55**, 377–388 (2023). 36823318 10.1038/s41588-023-01300-6 PMC10011140

[40] Teng, L., He, B., Wang, J. & Tan, K. 4DGenome: a comprehensive database of chromatin interactions. *Bioinformatics* **31**, 2560–2564 (2015). 25788621 10.1093/bioinformatics/btv158 PMC4514924

[41] Wang, Y. et al. The 3D Genome Browser: a web-based browser for visualizing 3D genome organization and long-range chromatin interactions. *Genome Biol.* **19**, 151 (2018). 30286773 10.1186/s13059-018-1519-9 PMC6172833

[42] Lopes, K. P. et al. Genetic analysis of the human microglial transcriptome across brain regions, aging and disease pathologies. *Nat. Genet.* **54**, 4–17 (2022). 34992268 10.1038/s41588-021-00976-y PMC9245609

[43] Nott, A. et al. Brain cell type-specific enhancer-promoter interactome maps and disease-risk association. *Science* **366**, 1134–1139 (2019). 31727856 10.1126/science.aay0793 PMC7028213

[44] Cifello, J. et al. hipFG: high-throughput harmonization and integration pipeline for functional genomics data. *Bioinformatics* **39**, btad673 (2023). 37947320 10.1093/bioinformatics/btad673 PMC10660288

[45] Kerimov, N. et al. A compendium of uniformly processed human gene expression and splicing quantitative trait loci. *Nat. Genet.* **53**, 1290–1299 (2021). 34493866 10.1038/s41588-021-00924-w PMC8423625

[46] Nasser, J. et al. Genome-wide enhancer maps link risk variants to disease genes. *Nature* **593**, 238–243 (2021). 33828297 10.1038/s41586-021-03446-x PMC9153265

[47] Selvarajan, I. et al. Integrative analysis of liver-specific non-coding regulatory SNPs associated with the risk of coronary artery disease. *Am. J. Hum. Genet.* **108**, 411–430 (2021). 33626337 10.1016/j.ajhg.2021.02.006 PMC8008493

[48] Burton, E.A. et. al. Variant‐to‐function mapping of late‐onset Alzheimer’s disease GWAS loci in human microglial models implicates RTFDC1 as an effector gene at the CASS4 locus. *Alzheimers Dement*. **20**(Suppl 1), 089683 (2025).

[49] Su, C. et al. 3D promoter architecture re-organization during iPSC-derived neuronal cell differentiation implicates target genes for neurodevelopmental disorders. *Prog. Neurobiol.* **201**, 102000 (2021). 33545232 10.1016/j.pneurobio.2021.102000 PMC8096691

[50] Littleton, S. H. et al. Variant-to-function analysis of the childhood obesity chr12q13 locus implicates rs7132908 as a causal variant within the 3’ UTR of FAIM2. *Cell Genom.* **4**, 100556 (2024). 38697123 10.1016/j.xgen.2024.100556 PMC11099382

[51] Moura, S. et al. Comparing Alzheimer’s genes in African, European, and Amerindian induced pluripotent stem cell-derived microglia. *Alzheimers Dement.* **21**, e70031 (2025). 40008916 10.1002/alz.70031 PMC11863361

[52] Akgun, B. et al. A genome-wide association study in Peruvians suggests new risk loci for Alzheimer disease. Preprint at *medRxiv*10.1101/2023.11.29.23299201 (2023).

[53] Mostafavi, H., Spence, J. P., Naqvi, S. & Pritchard, J. K. Systematic differences in discovery of genetic effects on gene expression and complex traits. *Nat. Genet.* **55**, 1866–1875 (2023). 37857933 10.1038/s41588-023-01529-1 PMC12270542

[54] Choi, H. J., Jeong, Y. J., Kim, J. & Hoe, H. S. EGFR is a potential dual molecular target for cancer and Alzheimer’s disease. *Front. Pharmacol.* **14**, 1238639 (2023). 37601068 10.3389/fphar.2023.1238639 PMC10433764

[55] Chiang, H. C., Wang, L., Xie, Z., Yau, A. & Zhong, Y. PI3 kinase signaling is involved in Abeta-induced memory loss in Drosophila. *Proc. Natl. Acad. Sci. USA* **107**, 7060–7065 (2010). 20351282 10.1073/pnas.0909314107 PMC2872421

[56] Fujita, M. et al. Cell subtype-specific effects of genetic variation in the Alzheimer’s disease brain. *Nat. Genet.* **56**, 605–614 (2024). 38514782 10.1038/s41588-024-01685-y PMC12288883

[57] Chiba-Falek, O., Gottschalk, W. K. & Lutz, M. W. The effects of the TOMM40 poly-T alleles on Alzheimer’s disease phenotypes. *Alzheimers Dement.* **14**, 692–698 (2018). 29524426 10.1016/j.jalz.2018.01.015 PMC5938113

[58] Leung, Y. Y. et al. Alzheimer’s Disease Sequencing Project release 4 whole genome sequencing dataset. *Alzheimers Dement.* **21**, e70237 (2025). 40407102 10.1002/alz.70237 PMC12100500

[59] Amlie-Wolf, A. et al. INFERNO: inferring the molecular mechanisms of noncoding genetic variants. *Nucleic Acids Res.* **46**, 8740–8753 (2018). 30113658 10.1093/nar/gky686 PMC6158604

[60] Carlson, C. S. et al. Selecting a maximally informative set of single-nucleotide polymorphisms for association analyses using linkage disequilibrium. *Am. J. Hum. Genet.* **74**, 106–120 (2004). 14681826 10.1086/381000 PMC1181897

[61] Hsu, F. et al. The UCSC known genes. *Bioinformatics* **22**, 1036–1046 (2006). 16500937 10.1093/bioinformatics/btl048

[62] Perez, G. et al. The UCSC Genome Browser database: 2025 update. *Nucleic Acids Res.* **53**, D1243–D1249 (2025). 39460617 10.1093/nar/gkae974 PMC11701590

[63] Jurka, J. Repbase update: a database and an electronic journal of repetitive elements. *Trends Genet.* **16**, 418–420 (2000). 10973072 10.1016/s0168-9525(00)02093-x

[64] Mudge, J. M. et al. GENCODE 2025: reference gene annotation for human and mouse. *Nucleic Acids Res.* **53**, D966–D975 (2025). 39565199 10.1093/nar/gkae1078 PMC11701607

[65] Sherry, S. T., Ward, M. & Sirotkin, K. dbSNP-database for single nucleotide polymorphisms and other classes of minor genetic variation. *Genome Res.* **9**, 677–679 (1999). 10447503

[66] Luo, Y. et al. New developments on the Encyclopedia of DNA Elements (ENCODE) data portal. *Nucleic Acids Res.* **48**, D882–D889 (2020). 31713622 10.1093/nar/gkz1062 PMC7061942

[67] Giambartolomei, C. et al. Bayesian test for colocalisation between pairs of genetic association studies using summary statistics. *PLoS Genet.* **10**, e1004383 (2014). 24830394 10.1371/journal.pgen.1004383 PMC4022491

[68] Sjostedt, E. et al. An atlas of the protein-coding genes in the human, pig, and mouse brain. *Science* **367**, eaay5947 (2020). 32139519 10.1126/science.aay5947

[69] Karlsson, M. et al. A single-cell type transcriptomics map of human tissues. *Sci. Adv.* **7**, eabh2169 (2021). 34321199 10.1126/sciadv.abh2169 PMC8318366

[70] Rentzsch, P., Witten, D., Cooper, G. M., Shendure, J. & Kircher, M. CADD: predicting the deleteriousness of variants throughout the human genome. *Nucleic Acids Res.* **47**, D886–D894 (2019). 30371827 10.1093/nar/gky1016 PMC6323892

[71] Dong, S. et al. Annotating and prioritizing human non-coding variants with RegulomeDB v.2. *Nat. Genet.* **55**, 724–726 (2023). 37173523 10.1038/s41588-023-01365-3 PMC10989417

[72] Sollis, E. et al. The NHGRI-EBI GWAS Catalog: knowledgebase and deposition resource. *Nucleic Acids Res.* **51**, D977–D985 (2023). 36350656 10.1093/nar/gkac1010 PMC9825413

[73] Greenfest-Allen, E. et al. NIAGADS Alzheimer’s GenomicsDB: A resource for exploring Alzheimer’s disease genetic and genomic knowledge. *Alzheimers Dement.* **20**, 1123–1136 (2024). 37881831 10.1002/alz.13509 PMC10916966

[74] Ramirez, A. M. et al. Ancestral genomic functional differences in oligodendroglia: implications for Alzheimer’s disease. *Alzheimers Dement.* **21**, e70593 (2025). 40937943 10.1002/alz.70593 PMC12426912

[75] Rao, S. S. et al. A 3D map of the human genome at kilobase resolution reveals principles of chromatin looping. *Cell* **159**, 1665–1680 (2014). 25497547 10.1016/j.cell.2014.11.021 PMC5635824

[76] Li, H. & Durbin, R. Fast and accurate long-read alignment with Burrows-Wheeler transform. *Bioinformatics* **26**, 589–595 (2010). 20080505 10.1093/bioinformatics/btp698 PMC2828108

[77] Lu, L. et al. Robust Hi-C maps of enhancer-promoter interactions reveal the function of non-coding genome in neural development and diseases. *Mol. Cell* **79**, 521–534.e515 (2020). 32592681 10.1016/j.molcel.2020.06.007 PMC7415676

[78] Zhang, S. et al. DeepLoop robustly maps chromatin interactions from sparse allele-resolved or single-cell Hi-C data at kilobase resolution. *Nat. Genet.* **54**, 1013–1025 (2022). 35817982 10.1038/s41588-022-01116-w PMC10082397

[79] Wingett, S. et al. HiCUP: pipeline for mapping and processing Hi-C data. *F1000Res* **4**, 1310 (2015). 26835000 10.12688/f1000research.7334.1 PMC4706059

[80] Langmead, B. & Salzberg, S. L. Fast gapped-read alignment with Bowtie 2. *Nat. Methods* **9**, 357–359 (2012). 22388286 10.1038/nmeth.1923 PMC3322381

[81] Labun, K. et al. CHOPCHOP v3: expanding the CRISPR web toolbox beyond genome editing. *Nucleic Acids Res.* **47**, W171–W174 (2019). 31106371 10.1093/nar/gkz365 PMC6602426

[82] Bae, S., Park, J. & Kim, J. S. Cas-OFFinder: a fast and versatile algorithm that searches for potential off-target sites of Cas9 RNA-guided endonucleases. *Bioinformatics* **30**, 1473–1475 (2014). 24463181 10.1093/bioinformatics/btu048 PMC4016707

