# Supplementary material for: Integrated genomic analysis and CRISPRi implicates EGFR in Alzheimer’s disease risk
Source: NPJ Dement. 2025 Dec 16;1(1):42. doi: 10.1038/s44400-025-00049-5 (PMC12711570; doi:10.1038/s44400-025-00049-5)
Supplement: Supplementary file 1 — Supplementary information [file 44400_2025_49_MOESM1_ESM.pdf]

Supplementary Figures

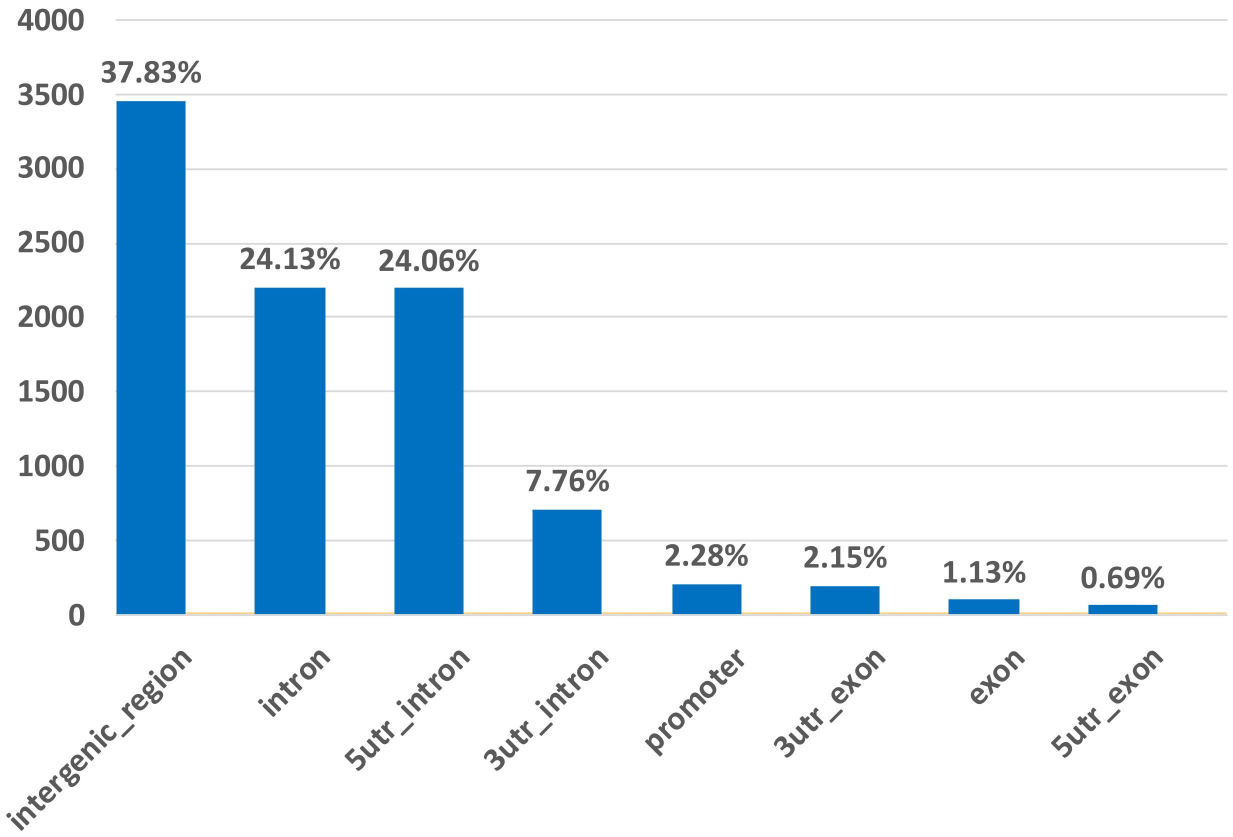

Supplementary Fig. 1: Genomic localization of candidate regulatory variants, shown as the proportion (%) in each category. Most (62%) are in intergenic and intronic regions, 35% in UTRs, and the rest in promoter and exonic regions.

A)

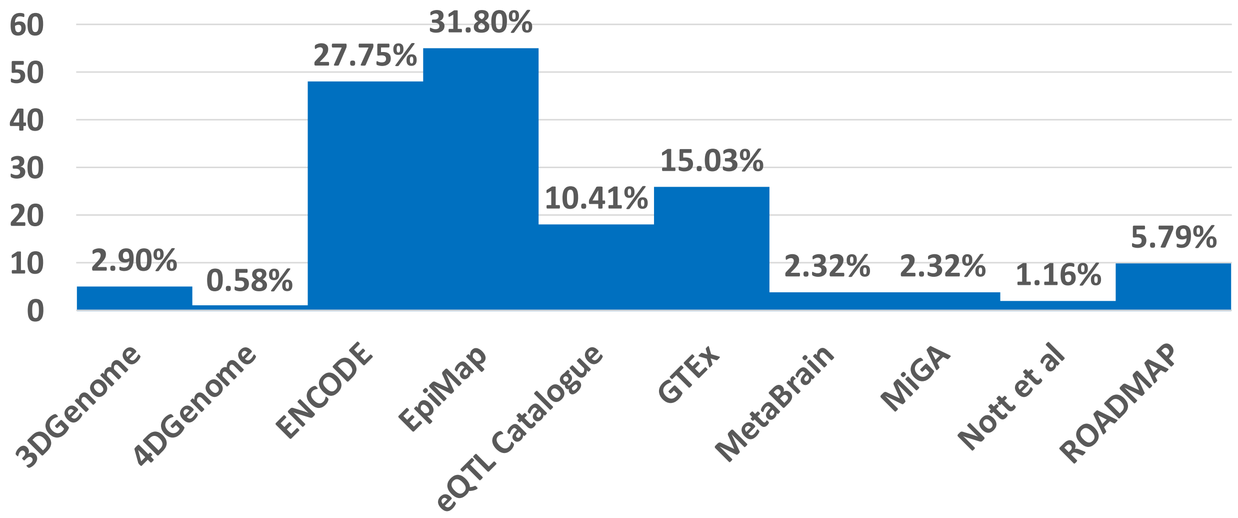

B)

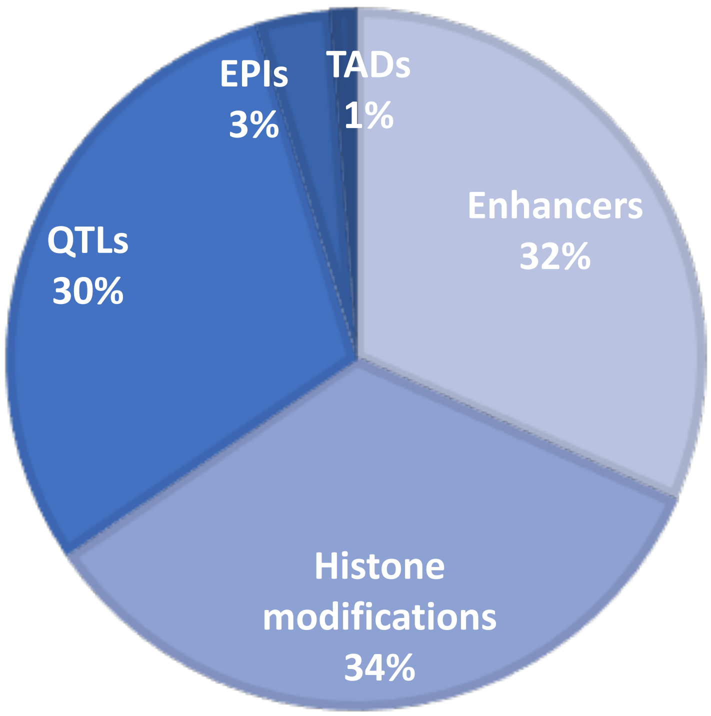

Supplementary Fig. 2: Breadth of the FILER brain related tracks used for this analysis. A) FILER brain related tracks by data sources. **B)** FILER brain related tracks by regulatory types.

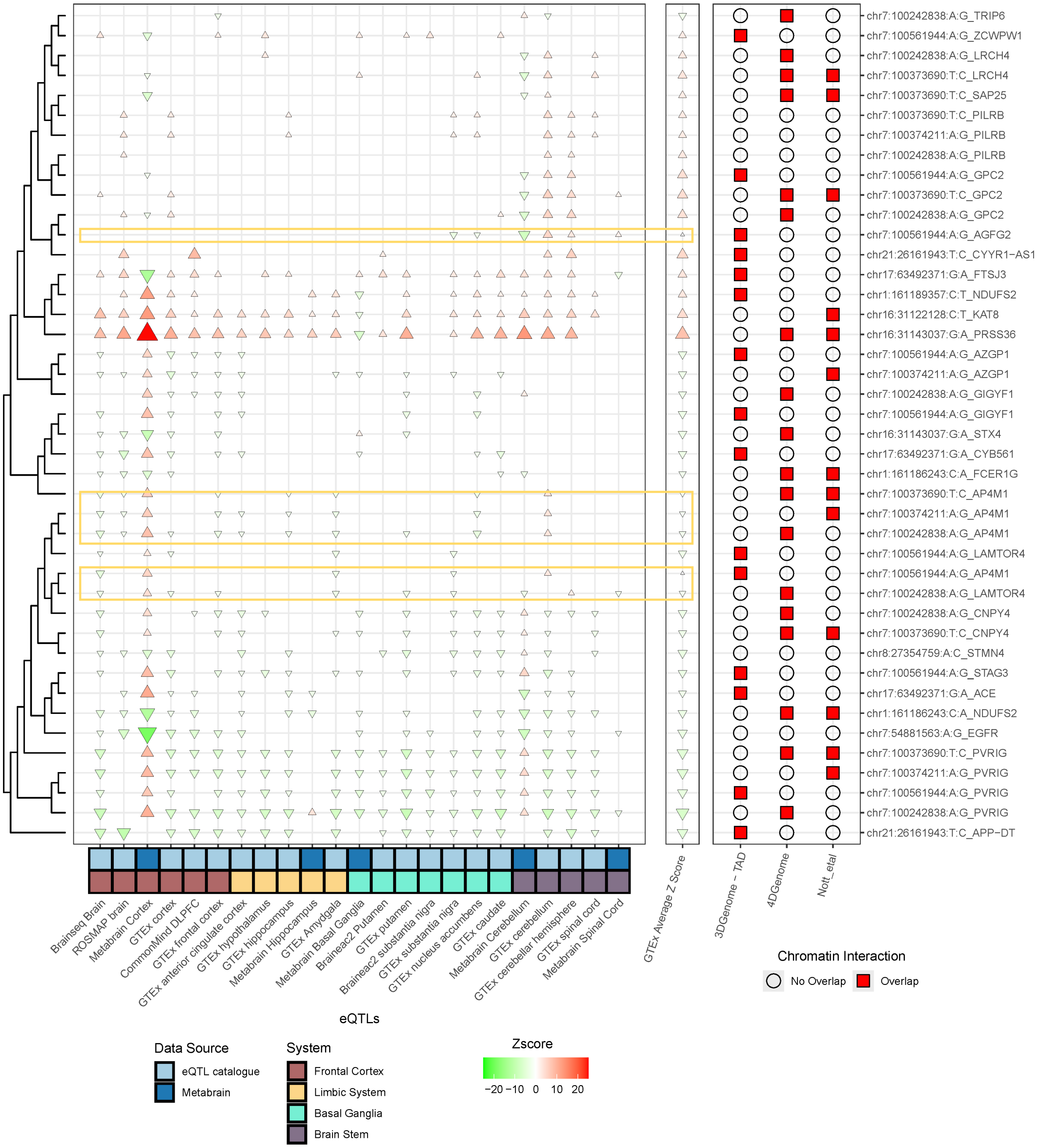

Supplementary Fig. 3: Comparison of directionality of selected putative V2G pairs across QTLs and EPI datasets used for *in silico* validation. Values in this plot are the original, non-normalized Z-scores (and therefore may be different from values in **Fig. 3**).

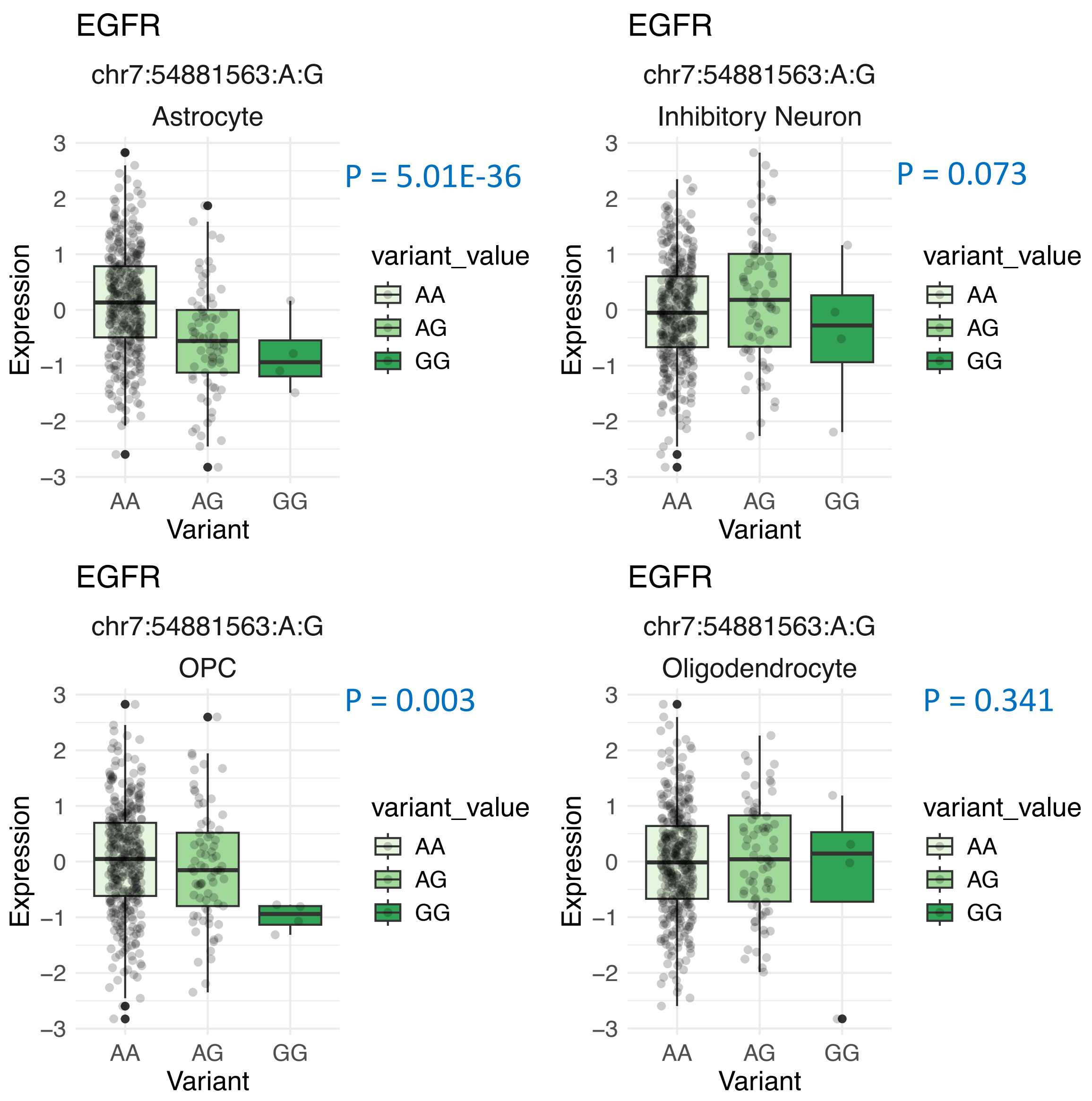

Supplementary Fig. 4. Single nuclei eQTL of rs74504435 and *EGFR* expression in four cell types (Astrocytes, Inhibitory Neurons, Oligodendrocyte precursor cells (OPCs), Oligodendrocytes) from ROSMAP.

Supplementary Tables

Supplementary Data 1 Number of regions, variants and genes identified in each of the four steps in **Fig. 1**.

Supplementary Data 2 Colocalization results with different PP.H4 levels used.

Supplementary Data 3 Significant colocalization results (with FDR<0.05 / coloc_PP.H4.abf > 0.95) identified in our analyses.

Supplementary Data 4 Colocalization results with different LD pruning thresholds.

Supplementary Data 5 Colocalization results with brain enhancers and predicted HOMER TFBS support.

Supplementary Data 6 Functional genomics datasets used for chromatin interaction analyses.

Supplementary Data 7 Chromatin interaction analyses results for the top four V2G pairs shown in **Fig. 4** (excluding chr7:54881563:A:G_EGFR, which was validated by CRISPRi).

Supplementary Data 8 Full list of candidate variants obtained from our "Top variant only Analyses" approach using the *Bellengeuz et al.* summary stats.

Supplementary Data 9 Full list of candidate variants obtained from our "Genome-wide Analyses" approach using the *Bellengeuz et al.* summary stats.

Supplementary Data 10 Full list of colocalization results (with target genes) obtained from our "Top variant only Analyses" approach using the *Bellengeuz et al.* summary stats.

Supplementary Data 11 Full list of colocalization results (with target genes) obtained from our "Genome-wide Analyses" approach using the *Bellengeuz et al.* summary stats.

Supplementary Data 12 FILER tracks used for functional genomic annotations.
